# Transcriptomic and metabolomic profiling of the potato plant response to zebra chip disease

**DOI:** 10.1371/journal.pone.0328035

**Published:** 2025-07-09

**Authors:** Margaret A. Carpenter, Tonya J. Frew, Helen L. Boldingh, Simona Nardozza, Martin L. Shaw, Susan J. Thomson, Rebecca D. Cooper, Gail M. Timmerman-Vaughan

**Affiliations:** 1 The New Zealand Institute for Plant and Food Research Limited, Christchurch, New Zealand; 2 The New Zealand Institute for Plant and Food Research Limited, Hamilton, New Zealand; 3 The New Zealand Institute for Plant and Food Research Limited, Auckland, New Zealand; University of Saskatchewan College of Agriculture and Bioresources, CANADA

## Abstract

Zebra chip disease of potato is caused by a bacterial pathogen, ‘*Candidatus* Liberibacter solanacearum’, vectored by the tomato potato psyllid (*Bactericera cockerelli* Sulc.). The plant response to the disease was explored using a combined transcriptomic and metabolomic approach. The effects of the disease were greater in tuber than in leaf or stem tissues, and represent a massive reprogramming of the tuber metabolism, with expression changes observed for many genes. In the tuber, starch synthesis was severely disrupted, with reduced expression of most starch synthesis genes, but increased expression of the gene encoding vacuolar invertase. This was consistent with increased glucose and fructose and reduced starch in the tuber, which are the hallmarks of the disease and the causes of the symptoms problematic to the potato industry. The phenylpropanoid pathway was more active in diseased tubers, as shown by increased transcript accumulation for phenylalanine ammonia lyase, cinnamate-4-hydroxylase, 4-coumarate:CoA ligase and cinnamyl alcohol dehydrogenase, and increased quantities of hydroxycinnamic acid amides, phenolic acids and coumarins. The expression of several genes encoding patatin storage proteins in the tuber was also decreased. In addition to the carbohydrate changes which cause undesirable visual symptoms associated with frying, the diseased tubers showed detrimental changes in nutritional value, such as increased toxic glycoalkaloids and decreased ascorbic acid.

## Introduction

Zebra chip (ZC) is a relatively new plant disease which is having a huge impact on potato (*Solanum tuberosum* L.) production in New Zealand, the USA, Mexico and Central America [1]. It decreases both the yield and quality of tubers produced and is resulting in huge losses in New Zealand potato farms [2]. The main concern is that when infected tubers are cut up and deep fried, they develop an unappealing dark colouration as a result of the Maillard reaction acting on amino acids and reducing sugars in the tuber tissue [3]. Therefore the major impact has been on potatoes grown for crisps and fries; however, the loss of quality also affects the fresh potato market. The disease causes changes in the taste of affected tubers but is not thought to pose a risk to human health [1]. Infected tubers are not suitable for seed, as they produce slender, weak sprouts or may fail to sprout [4]. Other symptoms include necrotic flecking around the vascular tissue in the tuber, increased enzymatic browning of tubers, upward rolling and discolouration of leaves, and stunted growth [5].

ZC is caused by the phloem-limited, unculturable, α-proteobacterium ‘*Candidatus* Liberibacter solanacearum’ (Lso), which is transmitted by an insect vector, tomato potato psyllid (TPP, *Bactericera cockerelli* Sulc*.*). TPP feeds on phloem sap from leaves, petioles and stems, using piercing-sucking mouthparts, and lays eggs on leaves, usually on the underside. TPP primarily lives on solanaceous plants including crops such as tomatoes, capsicums and tamarillos (*Solanum betaceum*), where it is a pest, and on wild plants such as nightshade and poroporo (*Solanum aviculare*) [6]. Lso causes disease in solanaceous crops involving chlorotic apical growth and yield losses, and also infects carrots and celery, where it is vectored by other psyllid species (*Trioza apicalis* and *Bactericera trigonica*) [7]. Lso infection appears to follow the movement of phloem sap in the plant from source to sink, i.e., from mature leaves to developing leaves and tubers [8] and from infected seed tubers into the sprouts as they grow [4]. Symptoms typically appear in potato plants 2–3 weeks after infection, at which time tuber development ceases [9]. The original identification of Lso was carried out by amplification and sequencing of 16S rRNA [10], which revealed a strong similarity to ‘*Candidatus* Liberibacter asiaticus’, the causal agent of citrus greening disease (also known as huanglongbing). The ZC pathosystem is similar to that of citrus greening which involves a Liberibacter pathogen, a psyllid vector, and a citrus species [8]. Polymerase chain reaction (PCR) assays are effective for detection of Lso in plant and psyllid tissues [11,12]. Lso can be detected in potato leaves 2–3 weeks after infection [13] and occurs in all parts of the plant [12].

The problematic symptoms of ZC that result from the Maillard reaction and the disruption of carbohydrate metabolism are only a small part of the biochemical changes which occur during the disease. ZC also causes increases in phenolics, peroxidases and polyphenol oxidases (PPO) in the tuber [3,14,15] and changes in starch, proteins and phenolics in the stem [16]. It has been suggested that stems are reprogrammed by the disease to exhibit tuber properties such as increased concentrations of starch and the patatin tuber storage proteins [16]. Transcriptomic studies have revealed transcriptional changes for genes associated with defence responses, starch metabolism, hormonal pathways, and photosynthesis, in both leaves and roots [17,18]. It has been suggested that ZC disease symptoms are largely due to malfunction of phloem loading, transport and unloading [8].

ZC is primarily being managed by using chemical insecticides to control the vector, or alternative methods such as agricultural oils [19], covering crops with mesh to exclude insects, and biological control [7]. There is much interest in breeding for ZC resistance, which would provide a preferable method of managing the disease; however, this is being hampered by a scarcity of resistant germplasm [20,21]. Progress has been made in breeding for tolerance to the disease, whereby infected plants show reduced tuber symptoms [22,23]. Reduction of the Maillard reaction products has also been achieved through invertase silencing [24]. Improved understanding of the genes, biochemical pathways and metabolites involved in the interactions between plant, pathogen and vector has potential to inform enhanced management of the disease as well as breeding for resistance/tolerance.

In this study we explored the effects of Lso infection on the potato plant transcriptome and metabolome to develop a comprehensive understanding of the response of the susceptible cultivar ‘Crop13’ (marketed as Moonlight) to Lso infection. Potato plants were infected with Lso via exposure to TPP. Leaf, stem and tuber samples from infected and control plants were characterised using RNA sequencing for transcriptomics and liquid chromatography mass spectrometry (LCMS) for untargeted metabolomics. The concentrations of sugars and starch, and the activity of the acid invertase enzyme, were also determined to clarify the changes in carbohydrate metabolism associated with ZC [9,18]. The results provide a global picture of the plant response to Lso infection, with the most substantial changes observed in the tuber.

## Methods

### Plant material and treatments

Potato plants of the susceptible ‘Crop13’ (marketed as Moonlight) [22] were grown from seed tubers in a glasshouse trial, using 4.8-L poly bags of potting mix, during spring 2014. Each plant was enclosed in an insect-proof mesh tent to facilitate TPP treatments and to prevent exposure to other insects. The trial was laid out in a complete randomised block design with four blocks, and with treatment levels Latinised across the rows. Plants were infected with Lso via exposure to infected TPP (denoted HP for “hot psyllid”). The TPP were from a colony grown on tomato plants which carried NZ1, a haplotype A strain of Lso [25]. Control plants were exposed to psyllids from an uninfected colony (CP for “cold psyllid”) or untreated (C for “control”). Three biological replicates of each treatment were grown. Plants were exposed to psyllids by introducing a tube containing 10 psyllids into the mesh tent when the plant was 35–40 cm tall (17 days after planting). After 2 days, plants were sprayed with Avid® (Syngenta, Basel, Switzerland) to kill the psyllids. The plants were then grown, with regular watering, for 7 weeks to provide time for the disease to develop.

Seven weeks post-treatment, leaf, stem and tuber samples were collected. Leaves from the middle of the plant were sampled, snap frozen in liquid nitrogen, crushed, and aliquoted into 5-mL tubes. Stem samples were taken from the middle of the stem, sliced with a scalpel into 3-mm slices, and snap frozen in liquid nitrogen. Tubers were removed from the potting mix, washed, then the largest tuber was sampled. Slices were cut from the stolon end of the tuber, and cut into 3-mm cubes, then snap frozen in liquid nitrogen. Samples were stored at −80°C for further analysis.

### Detection of Lso in tissue by qPCR

Samples (approximately 100 mg) of leaf and tuber in 2-mL microfuge tubes were freeze-dried for 2 days, then pulverized with a bead-beater (Tissuelyser II, Qiagen, Hilden, Germany) using 3-mm tungsten carbide beads. DNA was extracted using the Plant DNeasy® kit (Qiagen) following the manufacturer’s protocol. The Lso copy number of each sample was determined by qPCR following the method of Beard *et al*. [11] adapted for use with the Roche LightCycler® 480 (Roche, Basel, Switzerland) and PerfeCTa® SYBR® Green SuperMix® (Quantabio, Beverly, MA, USA). A section of the potato elongation factor 1α (EF1α) gene was also amplified, to allow the Lso copy number to be normalised. The Lso ratio was calculated as the Lso copy number divided by the EF1α copy number. The qPCR was performed on three biological replicates and three technical replicates per sample.

### Transcriptome sequencing

RNA was extracted from leaf and stem samples by grinding 100 mg of tissue in liquid nitrogen and using the Plant Virus RNA Kit (Geneaid, New Taipei City, Taiwan) according to the manufacturer’s instructions. As tuber tissue contains little RNA, a larger-scale method was used. Tuber tissue (1 g) was ground in liquid nitrogen and RNA extracted using the PureLink® Plant RNA Reagent (Thermo Fisher Scientific, Waltham, MA, USA) kit following the manufacturer’s large-scale protocol. Tuber RNA was re-suspended in 100 μL water, then further purified using RNeasy® MinElute spin columns (Qiagen). RNA samples were examined using a Fragment Analyzer (Agilent Technologies, Palo Alto, CA, USA) with the DNF-472 High Sensitivity RNA Analysis Kit (Agilent Technologies) to check integrity prior to constructing libraries, ensuring that RNA quality numbers were > 7.0 for tuber and stem RNA and > 6.0 for leaf RNA.

Transcriptome sequencing libraries were constructed following the method of Zhong *et al*. [26]. Barcoded libraries were pooled in three groups such that each of the three biological replicates was in a different pool. The libraries were sequenced at the Australian Genome Research Facility Ltd (Parkville, VIC, Australia) using an Illumina HiSeq 2000 (Illumina, San Diego, CA, USA), with two lanes per pool, generating 100-bp paired-end reads.

### Transcriptome data analysis

Raw Illumina reads were initially checked using FastQC v0.11.2 [27] and Fastq-screen v0.5.2 [28] for read quality and contaminants including potato virus Y and S. Data were trimmed using Trimmomatic v0.36 [29] to remove adapter and low quality sequences with basic trim settings, and also to remove any trimmed reads less than 50 bases in length: “HEADCROP:10 SLIDINGWINDOW:5:20 MINLEN:50”. Trimmed data were mapped to the Potato Genome Sequencing Consortium (PGSC) reference sequence (v4.03) [30–32] using the STAR v2.5.2a aligner in two-pass mode, along with the PGSC annotation version v3.4. Stranded counts were generated using HTseq v0.6.1 [33].

Differentially expressed genes were identified using the R package ‘DESeq2’ v1.22.2 [34]. The counts from the three tissues were analysed separately using pairwise comparisons between treatments. Genes were considered to be differentially expressed when the adjusted *p* value (padj) was < 0.001, and the absolute value of the log_2_ fold change was > 1. Functional annotation of the genes was based on the file PGSC_DM_v3.4_g2t2c2p2func_nonredundant.txt.zip downloaded from the PGSC website [31]. For some genes, the functional annotation was amended based on cited publications. The revised annotations are listed in S1 Table, which gives PGSC transcript ID numbers and functional annotation for all PGSC transcripts mentioned in the results and discussion. Functional relationships among differentially expressed genes were explored using KEGG pathway maps [35]. Gene ontology (GO) enrichment analysis was performed using the R package ‘topGO’ v2.34.0 [36] with Fisher’s exact test and the weight01 algorithm. The gene universe used for each tissue was the set of genes which had more than one sequence read aligned for that tissue, and also had GO annotation. The potato GO annotations used were produced using an ensemble of annotation pipelines to give a combined result, based on two or more of six pipelines agreeing [37]. Transcriptome sequences were deposited in the NCBI’s Sequence Read Archive under the BioProject ID PRJNA633718.

### Soluble acid invertase enzyme activity assay

Soluble acid invertase activities were assayed as previously described [38,39] with some modifications as described below. All samples were ground to a fine powder in liquid nitrogen. Aliquots of frozen powder (20 mg fresh weight) were extracted by vigorous vortexing with 500 μL of ice cold extraction buffer containing 5 mg of polyvinylpolypyrrolidone. The extraction buffer contained: 50 mM Hepes/KOH pH7.5, 10 mM MgCl_2_, 1 mM EDTA, 1 mM EGTA, 0.2 mM DTT, 1% v/v Triton X-100, 20% v/v glycerol and 0.25% w/v BSA protease free. The protease inhibitors used in [38] were replaced by one tablet of cOmplete™ ULTRA mini EDTA-free (Roche Diagnostics GmbH, Mannheim, Germany) per 10 mL of buffer. Crude extracts were not desalted but further diluted in ice cold extraction buffer to have a final 1500-fold dilution in the enzymatic assay(w/v) for tuber tissue and 2000-fold dilution (w/v) for leaf and stem tissues. Soluble acid invertase activities were assayed on a microplate at 25°C for 2 h in a volume of 50 μL. The reactions were started by adding 5 μL of diluted crude extracts or glucose standards (prepared in extraction buffer and ranging from 0 to 80 nM) to 45 μL of assay buffer (50 mM acetate/KOH, sucrose 20 mM and Triton X-100 0.3% (v/v)). The reaction was stopped by adding 10 μL of NaOH 0.5 M, followed by vortexing and 10 min of incubation at room temperature (21°C). The reaction was neutralised by adding 10 μL of HCl buffer (HCl 0.5 M and Hepes/KOH 0.1 M pH 7), followed by vortexing. Blank samples were stopped after 5 min. Glucose produced by sucrose cleavage was determined by fluorimetry (Victor3™ Multilabel Microplate Reader; Perkin Elmer, Waltham, Massachusetts, USA; excitation 540 nm and emission 570 nm; 30°C) after the addition of 50 μL of determination buffer, giving a final concentration in the reaction of 25 mM sodium phosphate buffer pH 7.4, 2 units/mL of glucose oxidase, 0.004 units/mL of horseradish peroxidase, and 0.2 mM dihydroxyphenoxazine.

### Carbohydrate analysis

Samples were ground in liquid nitrogen and subsamples of 0.2 g were weighed out. These were extracted in 80% ethanol (v/v) with fucose added as the internal standard, and then incubated for 1 h at 60°C. Extracted samples were centrifuged and the supernatant decanted off. The residue was re-suspended in 80% ethanol, re-spun and supernatants combined. The insoluble residue was transferred into Erlenmeyer flasks and analysed for starch [40]. A subsample of the supernatant was taken and dried using a centrifugal concentrator; samples were then re-dissolved in ultrapure water. The sugars were analysed using the DIONEX™ ICS-5000 Reagent-Free IC (RFIC; Thermo Fisher Scientific, Waltham, MA, USA) system with a CarboPac PA20 column and amino trap with electrochemical detection. Statistical analysis of the carbohydrate and invertase activity data was performed by analysis of variance using the R ‘stats’ package v3.5.1 [41] with tissue and treatment as factors. Pairwise comparisons between treatments were performed using the R package ‘emmeans’ [42] and standard errors of the means were calculated using ‘Rmisc’ [43].

### Untargeted metabolomics

Tissue samples (approximately 200 mg in a 2 mL tube) were freeze dried overnight, then powdered in a Tissuelyser II (Qiagen) at 25 Hz for 2 min, using 3-mm tungsten carbide beads. Aliquots of 4–10 mg were accurately weighed out and the weights recorded. Methanol (70% v/v) was added to achieve a final concentration of 10 mg/mL. The suspension was vortexed and spun at 12,000 g for 10 min, then 400 µL was filtered through a 0.2-μm polyvinylidene difluoride membrane into a low evaporation filter vial (Thomson Instrument Company, Oceanside, CA,USA) for direct injection.

The LCMS system consisted of a Thermo Scientific™ (San Jose, CA, USA) Q Exactive™ Plus Orbitrap coupled with a Vanquish™ Ultra-High Performance Liquid Chromatography system (Binary Pump H, Split Sampler HT, Dual Oven). It was calibrated immediately prior to each sample analysis batch with Thermo™ premixed solutions (Pierce™ LTQ ESI Positive and negative ion calibration solutions, catalogue numbers: 88322 and 88324, respectively).

Samples were analysed by four separate analytical methods (two chromatography systems, C18 (aqueous reversed phase) and Hydrophilic Interaction Liquid Chromatography (HILIC) (aqueous normal phase) with two ionisation modes -, + (n or p)) creating four datasets (Cn, Cp, Hn, Hp) which were analysed independently. For HILIC conditions, a 2-μL aliquot of each prepared extract was separated with a mobile phase consisting of 0.1% v/v formic acid in acetonitrile (A) and 5 mM ammonium acetate in water (B) by normal phase chromatography (Hypersil Gold HILIC 1.9 µm, 100 mm x 2.1 mm, P/N:26502–102130) maintained at 55°C with a flow rate of 400 µL/min. A gradient was applied: 0–1 min/5%B, linear increase to 12 min/98%B, isocratic 16 min/98%B, equilibration 16–17 min/5%B, isocratic to end 20 min/5%B.

For C18 conditions, a 2-μL aliquot of each prepared extract was separated with a mobile phase consisting of 0.1% formic acid in type 1 water (A) and 0.1% formic acid in acetonitrile (B) by reversed phase chromatography (Accucore™ Vanquish™ C18 1.5 µm, 100 mm x 2.1 mm, P/N: 27101–102130, Thermo Scientific) maintained at 40°C with a flow rate of 400 µL/min. A gradient was applied: 0–1 min/0%B, linear increase to 7 min/50%B, linear increase to 8 min/98%B, isocratic to 11 min/98%B, equilibration 11–12 min/0%B, isocratic to end 17 min/0%B.

The eluents from HILIC and C18 chromatography were scanned from 0.5 to 16 and 0.4 to 11.5 min respectively by API-MS (Orbitrap) with heated electrospray ionisation at 350°C in the negative and positive mode with capillary temperature of 320°C. Data were acquired for precursor masses from m/z 80–1200 amu (HILIC) and m/z 100–1500 amu (C18) at 70K resolution (AGC target 3e6, maximum IT 100 ms, profile mode) with data-dependent ms/ms for product ions generated by normalised collision energy (NCE:35, 45, 65) at 17.5K resolution (TopN 10, AGC target 2e5, Maximum IT 50 ms, Isolation 1.4 m/z).

Data were then processed with the aid of Xcalibur®4.1 and Compound Discoverer 3.0 (Thermo Electron Corporation). Calculated exact molecular weights generated from m/z ions and spectra features (isotope ratios, precursor and product fragment ions) were used to predict compound formula, with additional information from targeted search lists, internal and published library spectra and known compounds/metabolites. Differential analysis was applied based on grouping samples by treatment and tissue to identify compounds of interest. Significant features were manually interpreted or confirmed with reference to theoretical spectra features and/or literature from SciFinder™.

## Results

### Disease symptoms and detection of Lso in potato tissue by qPCR

**Fig 1:**
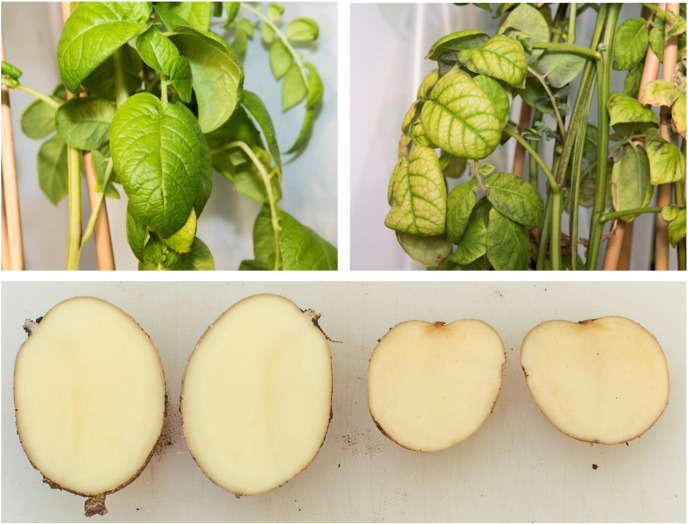
Leaves and tubers from plants from the CP (left) and HP (right) treatments. CP = uninfected tomato potato psyllid (TPP), HP = TPP +* *‘*Candidatus* Liberibacter solanacearum’ (Lso). The controls (C) were like CP.

When the plants were sampled at 7 weeks post-infection, the HP plants showed disease symptoms such as changes in leaf appearance (yellow, purple, or blotchy colouration), reduced tuber size, and increased browning of tuber tissue on exposure to air, particularly around the vascular ring and stolon attachment. In contrast, the tubers and leaves of the C and CP plants appeared healthy (Fig 1). The largest tubers produced by the HP plants were markedly smaller than those of the C and CP plants (S2 Table).

All potato leaf and tuber samples from C and CP plants tested negative for Lso via the Lso qPCR, whereas the HP potato plants gave positive results for both leaf and tuber samples (S3 Fig).

### Transcriptomic analysis of gene expression changes induced by Lso infection

After trimming, input sequence reads per sample ranged from 15,146,302–45,123,509 with a median read number of 33,004,482. Uniquely mapping reads ranged from 47% to 91% of total reads with a median unique mapping percentage of 83%, and with two samples falling below 60%. Of the 39,033 transcripts predicted from the potato genome, 27,072 were expressed in leaf samples, 26,706 in stem and 25,215 in tuber samples.

**Fig 2:**
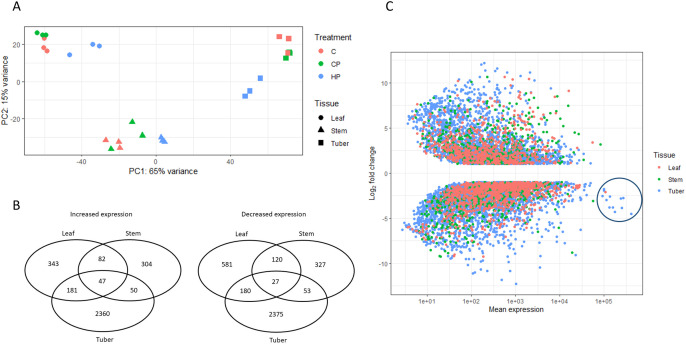
A. Principal component analysis of transcriptome count data. C = control, CP = uninfected tomato potato psyllid (TPP), HP = TPP +* *‘*Candidatus* Liberibacter solanacearum’ (Lso). B. **Venn diagrams of differentially expressed genes (DEGs) up- and down-regulated in each potato tissue for the HP vs CP contrast** (padj < 0.001 and log_2_ fold change > |1|). C. **Volcano plot showing log**_**2**_ **fold change and mean expression of differentially expressed genes (DEGs)** (padj < 0.001 and log_2_ fold change > |1|) from the HP vs CP contrast for three potato tissues. Mean expression is the mean number of read counts for all samples from each tissue. The circle defines a group of highly expressed genes downregulated by Lso infection.

In a principal component analysis of the transcriptome count data, the largest principal component (PC1) separated the transcriptomes by tissue. Within each tissue, there was clustering of replicates according to treatment (Fig 2A), with the samples from infected plants (HP) separate from the uninfected (C and CP) plant samples.

Differentially expressed genes (DEGs) were identified using pairwise comparisons among the HP, CP and C samples, with a threshold of padj < 0.001 and log_2_fold change > |1| used to define genes as differentially expressed. There were many DEGs, with tubers showing more DEGs than leaves or stems (Table 1). Relatively few DEGs were evident when CP samples were compared with C, which was expected as the psyllids were killed early in the experiment (2 days after infestation).

**Table 1:** Number of differentially expressed genes from comparisons between treatments of potato plants (padj < 0.001, log_2_ fold change > |1|. C = control, CP = uninfected tomato potato psyllid (TPP), HP = TPP +* *‘*Candidatus* Liberibacter solanacearum’ (Lso).

Comparison	Leaf	Stem	Tuber
**HP vs CP**	**1561**	**1010**	**5633**
**CP vs C**	**14**	**124**	**134**
**HP vs C**	**1583**	**2308**	**4418**

The HP vs CP contrast provides a focus on the effects of Lso on the plant, while controlling for the effects of the psyllid feeding, therefore the remainder of the transcriptome analyses presented below concern this contrast (S4-S6 Tables list the HP vs CP DEGs for each tissue). Of the DEGs revealed by the HP vs CP comparison, there were similar numbers of genes showing increases and decreases in expression (Fig 2B). There was relatively little overlap between tissues regarding the DEGs, indicating that each tissue was responding differently to the disease, with the tuber being more affected by the disease than either the leaf or stem.

Comparison of the HP vs CP DEGs (padj < 0.001 and log_2_ fold change > |1|) among the three tissues revealed that the tuber showed greater absolute fold changes then stem or leaf (Fig 2C) as well as more DEGs. The plot of log_2_ fold change vs mean expression (mean counts across all samples from each tissue) also revealed a group of highly expressed genes (circled in Fig 2C, base mean > 90,000) which showed a substantial negative fold change (log_2_ fold change <−1.8), indicating that they were highly expressed in the healthy growing tuber but repressed in the diseased tuber tissue. Of these, three encoded patatin proteins, four were associated with starch synthesis (sucrose synthase, glucose-1-phosphate adenylyltransferase (AGPase), a Kunitz type invertase inhibitor, and plastidic ATP/ADP-transporter), four were protease inhibitors (two aspartic protease inhibitors, serine protease inhibitor, metallocarboxypeptidase inhibitor), and the one showing the greatest decrease in expression encoded a lipoxygenase. The PGSC transcript ID numbers of these and other genes mentioned in the results and discussion are provided in S1 Table.

The genes showing the greatest absolute fold changes for each tissue are listed in Table 2, revealing the ten with the greatest increases, and the ten with the greatest decreases, in transcript accumulation in response to Lso infection. In leaves, the genes showing the greatest increases in expression included six heat shock proteins, whereas the genes showing the greatest decreases in expression included three auxin-induced Small Auxin-Up RNA (SAUR) genes. In stems, four genes involved in glycoalkaloid synthesis showed greatly increased expression, as did two genes encoding oxidoreductase, 2OG-Fe(II)oxygenase family proteins, while the genes with greatly decreased expression included those encoding four dirigent proteins (involved in stereochemistry of lignan synthesis) and linalool synthase (biosynthesis of linalool, an insecticidal volatile). In tubers, genes encoding two metal ion binding proteins, a Sugar Will Eventually be Exported Transporter (SWEET) sugar transporter and a dormancy-associated protein, were among the genes with the most increased expression, whereas those with large decreases in expression included two peroxidases and a patatin. Across all three tissues, there were major changes in expression of genes involved in redox reactions, cell wall structure and hormone signalling.

**Table 2:** Differentially expressed genes (DEGs)^a^ showing the greatest fold changes from each potato tissue: 10 with greatest increase in expression and 10 with greatest decrease in expression.

Transcript	Base Mean^b^	Log_2_ Fold Change	Adjusted p value	Annotation
**Leaf**				
PGSC0003DMT400078007	556	10.6	1.044E-09	17.6 kD class I small heat shock protein
PGSC0003DMT400008351	1979	10.4	3.718E-07	Small heat shock protein, chloroplastic
PGSC0003DMT400073689	846	10.3	2.944E-08	Small heat shock protein
PGSC0003DMT400054528	50	10.2	0.0001286	Conserved gene of unknown function
PGSC0003DMT400062882	758	9.7	9.736E-09	Heat shock protein
PGSC0003DMT400009069	15605	9.1	1.222E-12	Abscisic acid and environmental stress-inducible protein TAS14 (dehydrin)
PGSC0003DMT400080935	1252	8.9	2.116E-08	Peptidylprolyl isomerase (cyclophilin, protein folding)
PGSC0003DMT400078202	2225	8.9	5.901E-12	Hsp20.1 protein (heat shock)
PGSC0003DMT400023932	1209	8.6	8.954E-09	Small heat shock protein homolog protein
PGSC0003DMT400025150	48	8.6	3.153E-06	1-aminocyclopropane-1-carboxylate oxidase (redox)
PGSC0003DMT400042533	184	−9.3	1.234E-09	Conserved gene of unknown function
PGSC0003DMT400072585	41	−9.2	2.572E-07	Short chain alcohol dehydrogenase
PGSC0003DMT400007430	41	−9.0	1.644E-08	Transcription regulator
PGSC0003DMT400055827	27	−7.9	2.741E-05	Beta-expansin 3 (cell wall)
PGSC0003DMT400041726	141	−7.9	2.178E-09	Flowering locus T (tuberization, development)
PGSC0003DMT400034479	13	−7.6	1.115E-05	Auxin-induced SAUR
PGSC0003DMT400013351	50	−7.5	2.014E-05	Pectinesterase (cell wall)
PGSC0003DMT400012237	103	−7.5	5.255E-08	Conserved gene of unknown function
PGSC0003DMT400042692	9	−7.4	1.931E-06	Auxin-induced SAUR
PGSC0003DMT400034478	8	−7.1	0.0001474	Auxin-induced SAUR
**Stem**				
PGSC0003DMT400066846	228	10.6	2.147E-18	ERF1 (ethylene-responsive transcription factor, GAME9)
PGSC0003DMT400055111	98	10.1	1.398E-11	Oxidoreductase, 2OG-Fe(II) oxygenase family protein (redox)
PGSC0003DMT400030670	1706	8.8	6.376E-27	UDP-galactose:solanidine galactosyltransferase (glycoalkaloid synthesis, SGT1, GAME1)
PGSC0003DMT400030678	8769	8.5	1.361E-22	Cellulose synthase (cell wall)
PGSC0003DMT400008930	33	8.4	3.825E-09	Oxidoreductase, 2OG-Fe(II) oxygenase family protein (redox)
PGSC0003DMT400030650	2606	8.3	6.045E-25	SGA (glycoalkaloid synthesis, SGT3, GAME2)
PGSC0003DMT400064961	14	8.3	7.436E-08	Gamma aminobutyrate transaminase isoform2 (glycoalkaloid synthesis, GAME12)
PGSC0003DMT400026285	1978	8.1	2.677E-24	Cysteine protease inhibitor 1 (programmed cell death)
PGSC0003DMT400010306	84	8.0	3.835E-09	Anthranilate N-benzoyltransferase protein (phytoalexin biosynthesis)
PGSC0003DMT400053259	33	8.0	7.396E-06	Conserved gene of unknown function
PGSC0003DMT400077599	20	−9.3	6.829E-07	Conserved gene of unknown function (dirigent)
PGSC0003DMT400016340	19	−9.2	8.027E-08	Peroxidase (redox)
PGSC0003DMT400030271	29	−8.9	5.138E-07	Disease resistance-responsive family protein (dirigent)
PGSC0003DMT400026448	18884	−8.8	2.69E-08	Senescence-specific cysteine protease
PGSC0003DMT400040204	25	−8.6	2.274E-05	Conserved gene of unknown function (dirigent)
PGSC0003DMT400022230	12	−8.5	0.0001245	Disease resistance-responsive family protein (dirigent)
PGSC0003DMT400029275	16	−7.8	1.703E-06	Linalool synthase (terpenoid synthesis)
PGSC0003DMT400005637	84	−7.7	8.191E-06	Protein phosphatase-2c (abscisic acid signalling)
PGSC0003DMT400069577	45	−7.7	4.292E-06	UPF0497 membrane protein (CASP = casparian strip membrane domain protein)
PGSC0003DMT400041080	108	−7.6	4.805E-07	Abscisic acid receptor PYL4 (jasmonate signalling)
**Tuber**				
PGSC0003DMT400015350	194	12.2	3.721E-06	Metal ion binding protein
PGSC0003DMT400029104	165	12.0	6.17E-09	Heat shock protein hsp70
PGSC0003DMT400049728	377	11.6	1.35E-07	Cytochrome P450 hydroxylase
PGSC0003DMT400021966	204	11.4	1.786E-08	MtN3 protein (SWEET)
PGSC0003DMT400033385	345	11.3	8.548E-11	Auxin repressed/dormancy associated protein
PGSC0003DMT400058342	633	11.1	8.477E-12	UPF0497 membrane protein In26 (CASP)
PGSC0003DMT400031731	2090	11.1	7.334E-15	ATP binding protein
PGSC0003DMT400040055	1296	10.8	8.479E-08	Dehydrin DH2a (dehydration response)
PGSC0003DMT400001233	153	10.6	9.164E-08	Zinc finger protein
PGSC0003DMT400024481	52	10.4	6.174E-05	Metal ion binding protein
PGSC0003DMT400027955	1030	−12.3	4.883E-27	Pectate lyase (cell walls)
PGSC0003DMT400027953	477	−11.8	3.086E-20	Glutaredoxin (redox)
PGSC0003DMT400065498	108	−11.1	2.904E-17	Peroxidase 25 (redox)
PGSC0003DMT400029506	120	−11.0	1.692E-15	Flowering promoting factor-like 1
PGSC0003DMT400013504	750	−10.9	2.142E-18	Peroxidase (redox)
PGSC0003DMT400060932	227	−10.8	2.885E-17	Patatin B2
PGSC0003DMT400034984	163	−10.7	9.308E-17	AP2/ERF domain-containing transcription factor
PGSC0003DMT400074028	3563	−10.5	3.851E-51	Conserved gene of unknown function
PGSC0003DMT400090651	82	−10.2	1.435E-15	Dehydration-responsive element-binding protein 1B
PGSC0003DMT400079558	683	−9.9	3.904E-45	Polygalacturonase non-catalytic subunit AroGP3 (cell walls)

^a^DEGs are for the comparison between HP (tomato potato psyllid infected with ‘*Candidatus* Liberibacter solanacearum’) and CP (uninfected tomato potato psyllid).

^b^Base mean is mean number of read counts for all samples from each tissue.

### Differential expression of carbohydrate metabolism genes.

**Fig 3:**
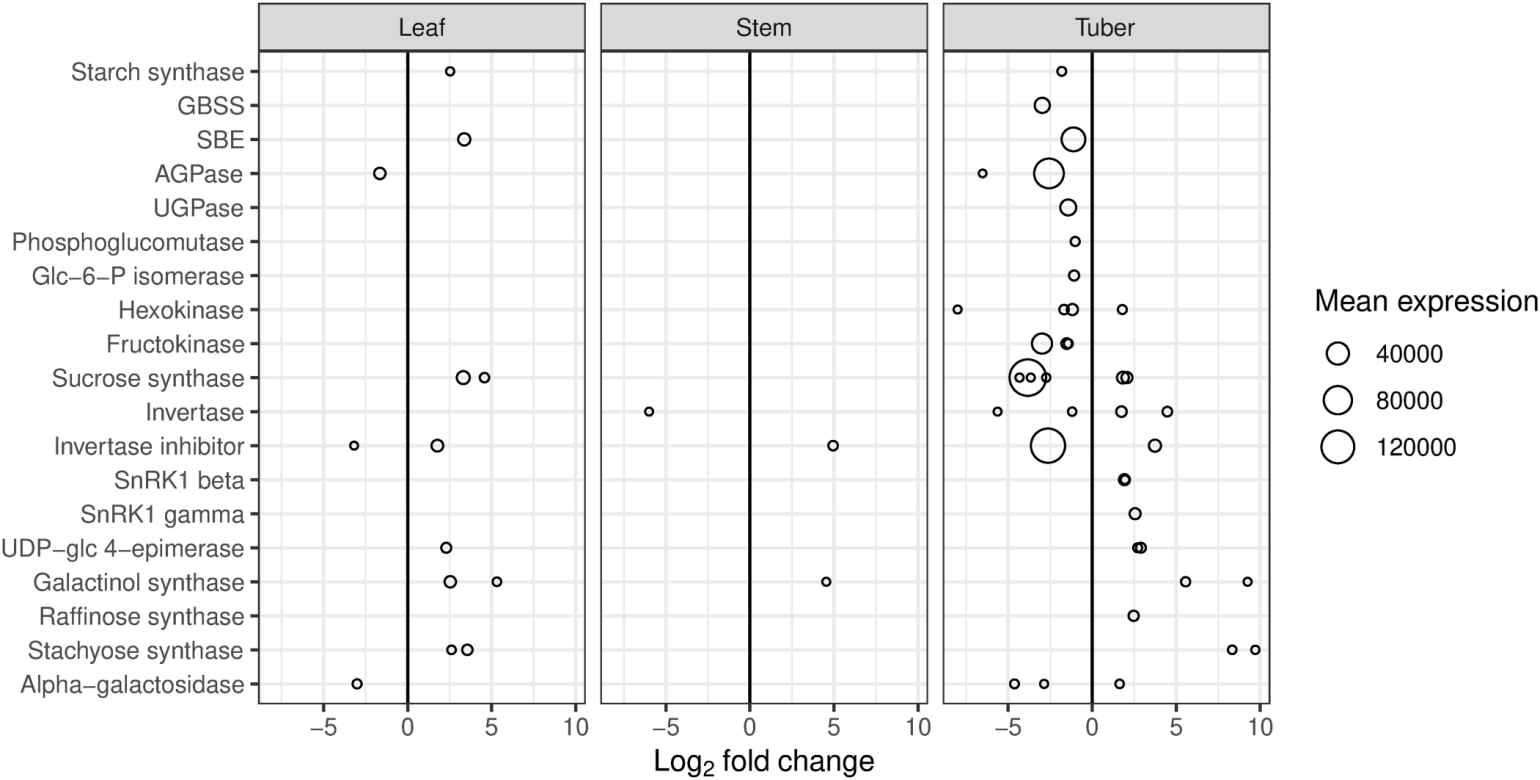
Differentially expressed genes (DEGs) involved in biosynthesis of starch and metabolism of raffinose family oligosaccharides. DEGs are for the comparison between HP (tomato potato psyllid infected with ‘*Candidatus* Liberibacter solanacearum’) and CP (uninfected tomato potato psyllid). Mean expression is the mean number of read counts for all samples from each potato tissue.

Infection with Lso affected the expression of genes involved in sucrose and starch metabolism in potato plants, particularly the starch synthesis pathway (Fig 3). In tuber tissue, many genes essential for starch synthesis showed decreased expression in HP samples compared with CP samples. These included genes encoding starch synthases and starch branching enzymes, as well as genes involved in substrate supply: AGPase, UTP-glucose-1-phosphate uridylyltransferase (UGPase), phosphoglucomutase (PGM), glucose-6-phosphate isomerase, hexokinase, fructokinase, and sucrose synthase. In some cases there were several genes with a common function that showed different levels of expression, and magnitudes and directions of change, but the combined result was a decrease in gene expression. In contrast, two invertase genes showed increased expression. Some of the genes involved in regulation of invertase activity were also differentially expressed: invertase inhibitor genes showed decreased expression whereas the genes encoding the β and γ subunits of sucrose nonfermenting1-related kinase (SnRK1) were increased. In leaf samples, some of the genes involved in starch synthesis showed increased expression during Lso infection (sucrose synthase, sucrose phosphate synthase, starch synthase, starch branching enzyme), whereas AGPase expression decreased. Stem samples did not show major changes in starch gene expression.

Marked changes were also observed in the genes involved in raffinose family oligosaccharide (RFO) metabolism. Galactinol synthase, raffinose synthase and stachyose synthase genes showed increased expression in the tuber, indicating an increase in synthesis of RFOs (Fig 3). Stachyose synthase expression was also increased in leaf tissue, while galactinol synthase expression was increased in all three tissues. Invertases are also involved in this pathway and as previously mentioned, invertase gene expression was increased in infected tubers. Genes encoding α-galactosidase, which catabolizes RFOs, were decreased in the leaf and tuber. UDP-glucose-4-epimerase gene expression, involved in galactinol synthesis, was increased in the leaf and tuber.

SWEETs are sugar transporters that transport di- or monosaccharides across membranes. The genes encoding 27 SWEETs have been identified in potato, and of these nine were differentially expressed in the tuber, three in the stem and one in the leaf (S1 Table). In all tissues, there were more SWEET genes showing increased expression than decreased expression, in response to Lso infection. Two SWEET genes were also among those showing the biggest fold changes in tuber tissue, with log_2_fold changes of 11.4 and 10.2. The gene encoding the sucrose transport protein SUT1 was also differentially expressed, with deceased expression in the tuber and increased expression in leaf tissue.

### Differential expression of genes involved in secondary metabolism.

**Fig 4:**
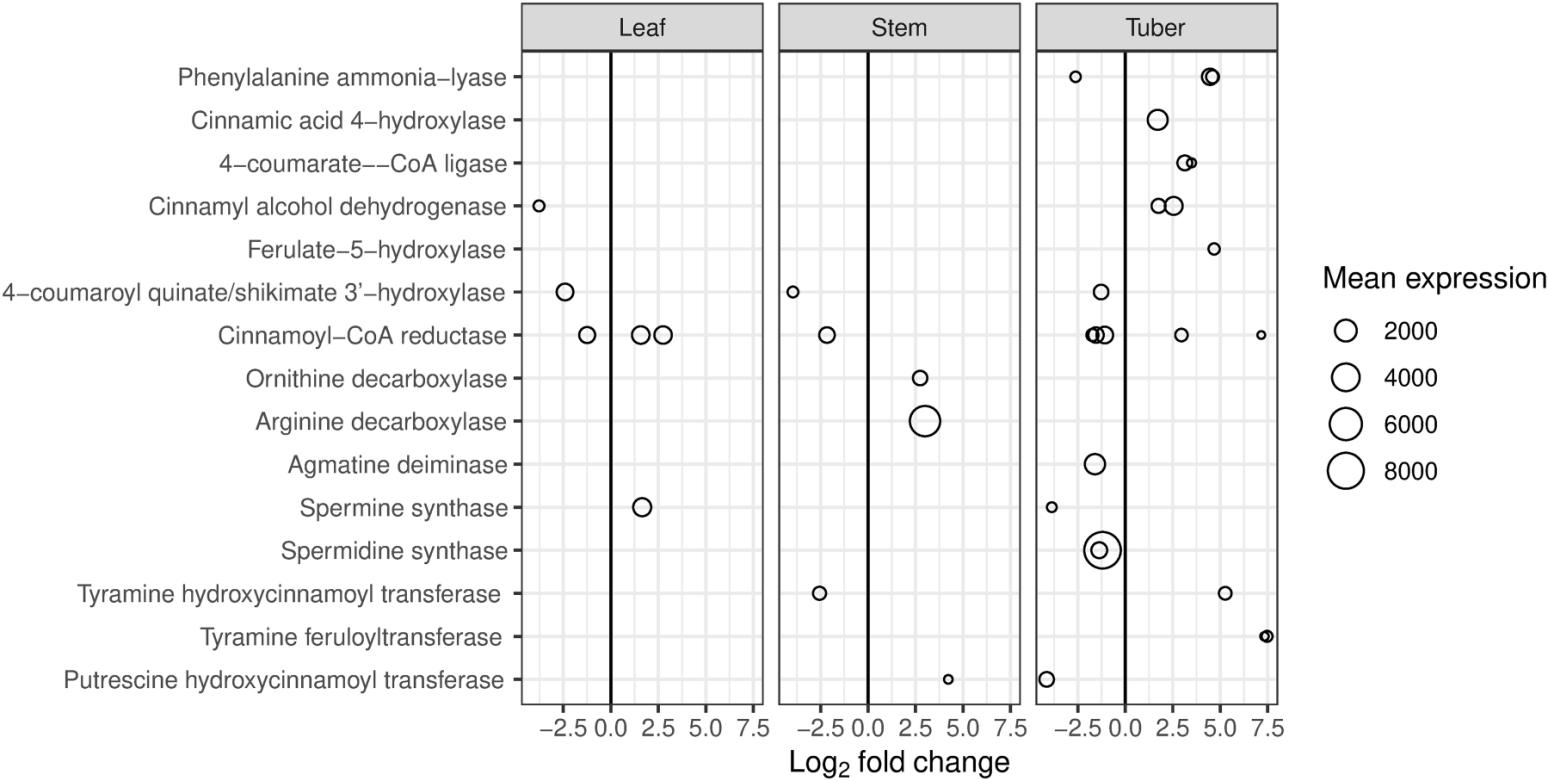
Differentially expressed genes (DEGs) in the phenylpropanoid pathway and hydroxycinnamic acid amide (HCAA) biosynthesis in potato. DEGs are for the comparison between HP (tomato potato psyllid infected with ‘*Candidatus* Liberibacter solanacearum’) and CP (uninfected tomato potato psyllid). Mean expression is the mean number of read counts for all samples from each tissue.

Several genes involved in phenylpropanoid biosynthesis showed increased expression (Fig 4). In the core phenylpropanoid pathway, the genes encoding phenylalanine ammonia lyase (PAL), cinnamate 4-hydroxylase and 4-coumaroyl:CoA ligase all showed significantly increased expression in the tuber, but not in the stem or leaf. Cinnamoyl alcohol dehydrogenase and ferulate 5-hydroxylase also showed increased expression in the tuber, whereas 4-coumaroyl quinate/shikimate 3’ hydroxylase had decreased expression and the cinnamoyl CoA reductase genes gave mixed results. Of the genes involved in polyamine biosynthesis, ornithine and arginine decarboxylases showed increased expression in the stem, while agmatine deiminase and spermine and spermidine synthases showed reduced expression in the tuber. Genes encoding two tyramine hydroxycinnamoyl transferases involved in hydroxycinnamic acid amide (HCAA) biosynthesis were differentially expressed: one showed an increase in tuber tissue while another showed a decrease in stem tissue. Putrescine hydroxycinnamoyl transferase, also involved in HCAA biosynthesis, showed increased expression in the stem and decreased expression in the tuber, while the expression of two tyramine feruloyltransferase genes was increased in tuber tissue.

Polyphenol oxidases are associated with enzymatic browning and in potato are encoded by 12 genes (including catechol oxidase and diphenol oxidases). Three showed decreased expression in the tuber, including one (PGSC0003DMT400048684) which was very highly expressed in healthy tubers but very weakly expressed in infected tubers (S1 Table). The genes encoding three laccases, which also oxidise phenolic compounds and are involved in lignification, showed increased expression.

Peroxidases are encoded by 137 genes in potato, of which 36 were differentially expressed in the tuber, 10 showing increased expression and 26 decreased expression (S1 Table). The peroxidase gene with the highest level of expression in tubers, encoding secretory peroxidase, showed a large decrease in expression in response to infection.

**Fig 5:**
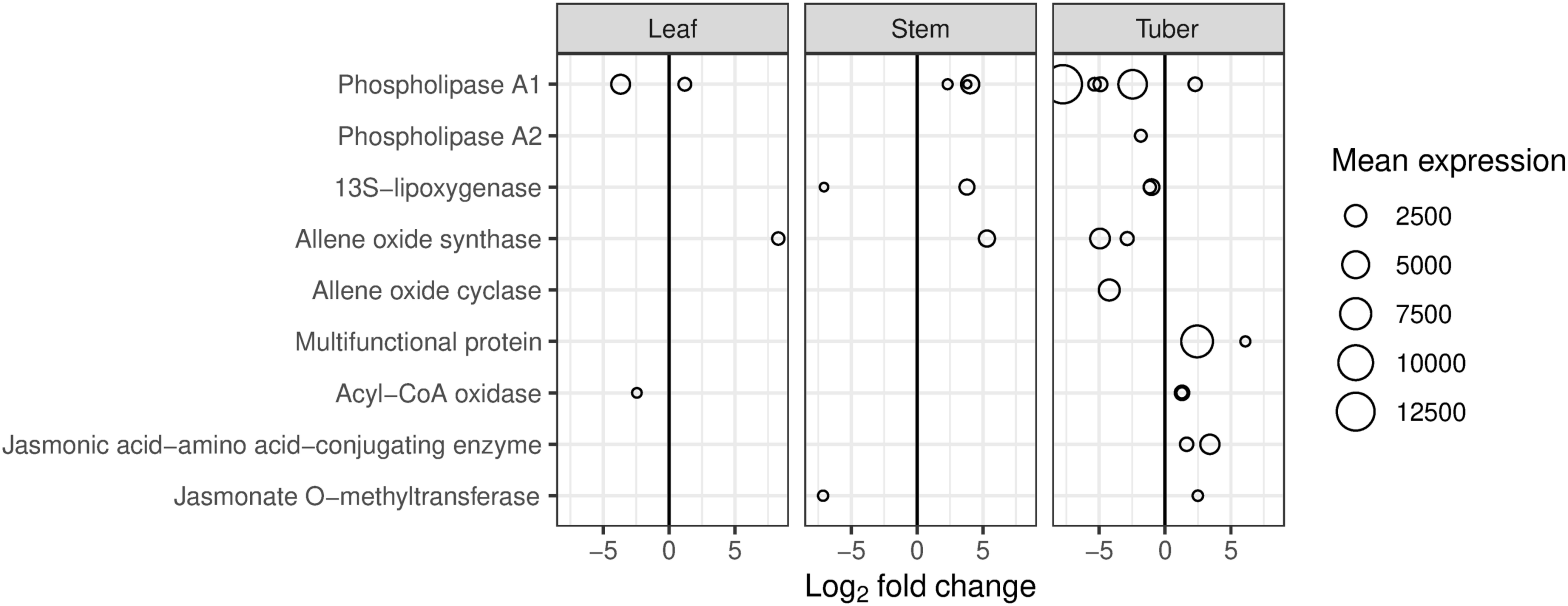
Differentially expressed genes (DEGs) involved in linolenic and jasmonic acid metabolism in potato. DEGs are for the comparison between HP (tomato potato psyllid infected with ‘*Candidatus* Liberibacter solanacearum’) and CP (uninfected tomato potato psyllid). Mean expression is the mean number of read counts for all samples from each tissue.

Genes involved in synthesis of linolenic and jasmonic acid (JA) showed contrasting expression patterns in tuber tissue. Of the transcripts involved in the JA precursor synthesis, two phospholipases (A1 and A2), a 13S-lipoxygenase, an allene oxide synthase, and an allene oxide cyclase showed decreased expression (Fig 5). However, genes encoding enzymes further down the pathway to JA, had increased expression (i.e., multifunctional protein, acyl-CoA oxidase). Additionally, the genes associated with biosynthesis of the JA derivatives methyl jasmonate and JA-isoleucine also showed increased expression in the tuber. In the leaf and stem, fewer of these genes were differentially expressed.

**Fig 6:**
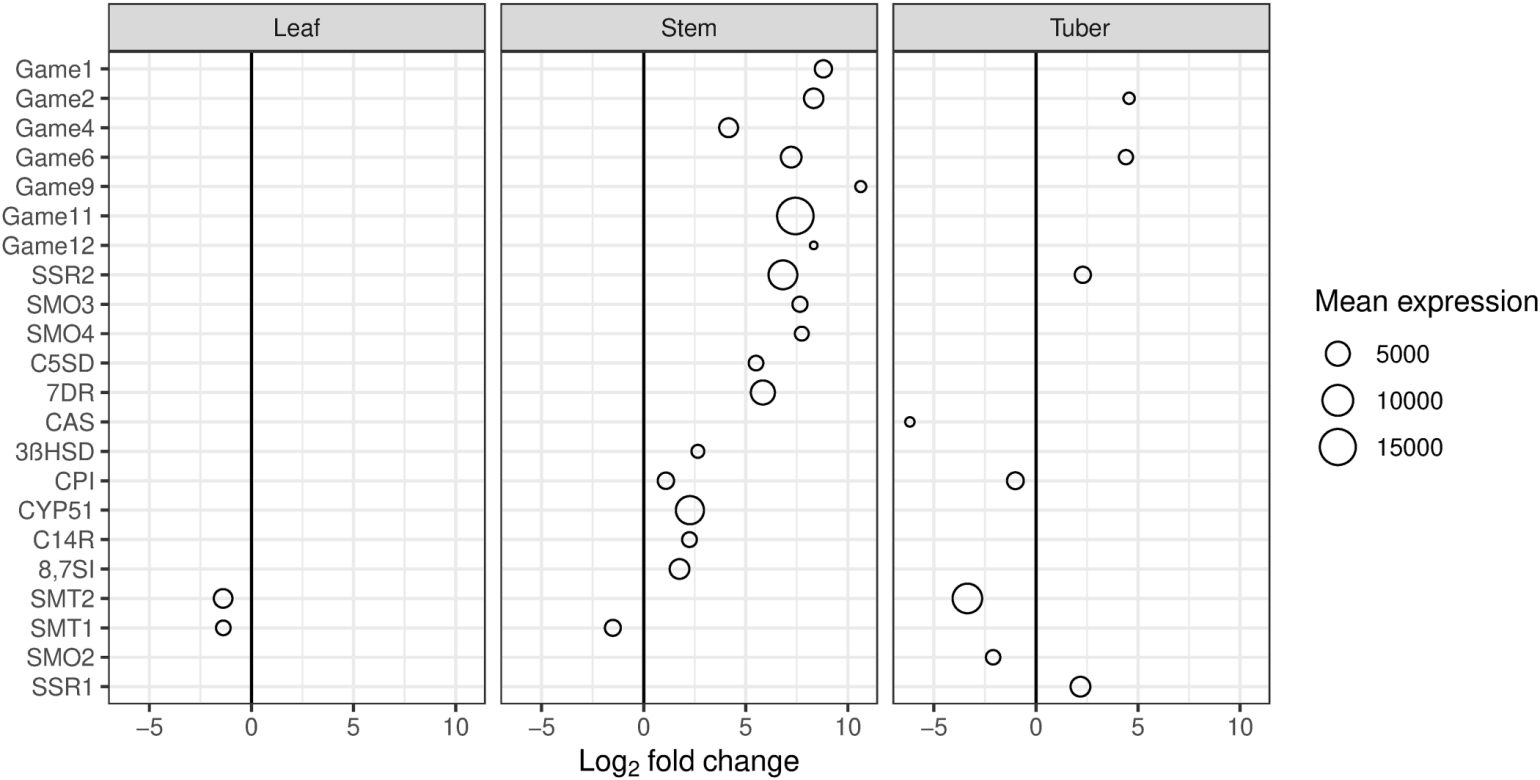
Genes involved in biosynthesis of steroidal glycoalkaloids showed increased expression in potato stem tissue in the HP (tomato potato psyllid infected with ‘*Candidatus* Liberibacter solanacearum’) samples, compared to the CP (uninfected tomato potato psyllid) samples. SGT1 = UDP-galactose:solanidine galactosyltransferase (Game1), SGA = UDP-glycosyl transferase (Game2), GABA = gamma aminobutyrate transaminase (Game12), ERF1 = ethylene responsive factor (Game9), CytP450 = Game 6 and Game 4, 2-ODD = 2-oxyglutarate-dependent dioxygenase (Game11), SSR2 = sterol side-chain reductase, SMO3 = sterol methyl oxidase 3, SMO4 = sterol methyl oxidase 4, C5SD = sterol C-5 [6] desaturase, 7DR = 7-dehydrocholesertol reductase, CAS = cycloartenol synthase, 3βHSD = 3β-hydroxysteroid dehydrogenase, CPI = cyclopropylsterol isomerase, CYP51 = sterol C-14 demethylase, C14R = sterol C-14 reductase, 8,7SI = sterol 8,7 isomerase, SMT2 = sterol methyl transferase 2, SMT1 = sterol methyl transferase 1, SMO2 = sterol methyl oxidase 2, SSR1 = sterol side-chain reductase 1. Game = Glycoalkaloid metabolism genes [44]. Mean expression is the mean number of read counts for all samples from each tissue.

Genes involved in the biosynthesis of sterols and steroidal alkaloids showed substantial increases in expression in the stem, but few showed changes in tuber and leaf tissues (Fig 6). Specifically, several genes in the biosynthetic pathway from cholesterol to steroidal alkaloids had increased expression in the stem (SGT1, SGA, ERF1, GABA transaminase, two cytochrome P450s and a 2-oxoglutarate dependent dioxygenase). The first four of these genes were among those with the greatest fold changes observed in stem tissue (Table 2). Genes involved in biosynthesis of cholesterol from cycloartenol were also increased in the stem (SSR2, SMO3, SMO4, C5SD, 7DR, 3βHSD, CPI, CYP51, 8,7SI). Some of these genes are also involved in sterol synthesis; however, other genes involved in sterol synthesis (SMO2, SMT1, SMT2, SSR1), which are not involved in steroidal alkaloid synthesis, did not have increased expression in the stem.

Many genes encoding heat shock proteins showed increased expression in response to infection in both leaf and stem tissues, with greater fold changes in the leaf than in the stem (S1 Table). In leaf tissue, 31 genes encoding heat shock proteins showed increased expression, while two showed decreased expression. In stem tissue, 27 were increased and none decreased. There were also some genes encoding heat shock proteins with increased expression in the tuber (n = 16) but a similar number [15] with decreased expression, and the mean expression levels of the DEGs were lower in the tuber than in the leaf and stem.

In potato, there are four genes which encode tuber-specific and sucrose responsive element binding factors. Of these, three showed deceased expression in the tuber, while one showed increased expression in the leaf (S1 Table).

Patatins are the major storage proteins in potato tubers, and are encoded by 15 genes. Of these, nine were differentially expressed in tuber tissue, and all nine showed decreased expression in response to Lso infection (S1 Table). This included three of the genes with the highest mean expression in the tuber (Fig 2C). Only three patatin genes were differentially expressed in the leaf and stem, and these showed increased expression in both tissues.

### Gene ontology enrichment.

GO enrichment analysis was performed for the HP vs CP comparison for each tissue. GO terms related to redox and stress responses were enriched, with genes showing increased expression for all tissues. In tubers, transcription factor activity was increased, whereas GO terms associated with the microtubules, which have roles in growth, immune response and signalling, were enriched for genes showing decreased expression. In leaves, GO terms related to photosynthesis and protein phosphorylation were enriched for genes showing deceased expression. In stems, sterol biosynthesis genes showed increased activity, while genes associated with membranes and transport had decreased expression. Enrichment of hormone-related GO terms also occurred, with auxin-related genes having decreased expression in the leaf and stem, and gibberellin-related genes also decreased in the stem (indicating reduced growth), while in leaves expression of genes related to abscisic acid (ABA) signalling was increased, signifying responses to stress. GO terms significantly enriched at *p* < 0.01 are listed in S7 Table.

### Changes in carbohydrate concentrations and soluble acid invertase activity in response to Lso infection

**Fig 7:**
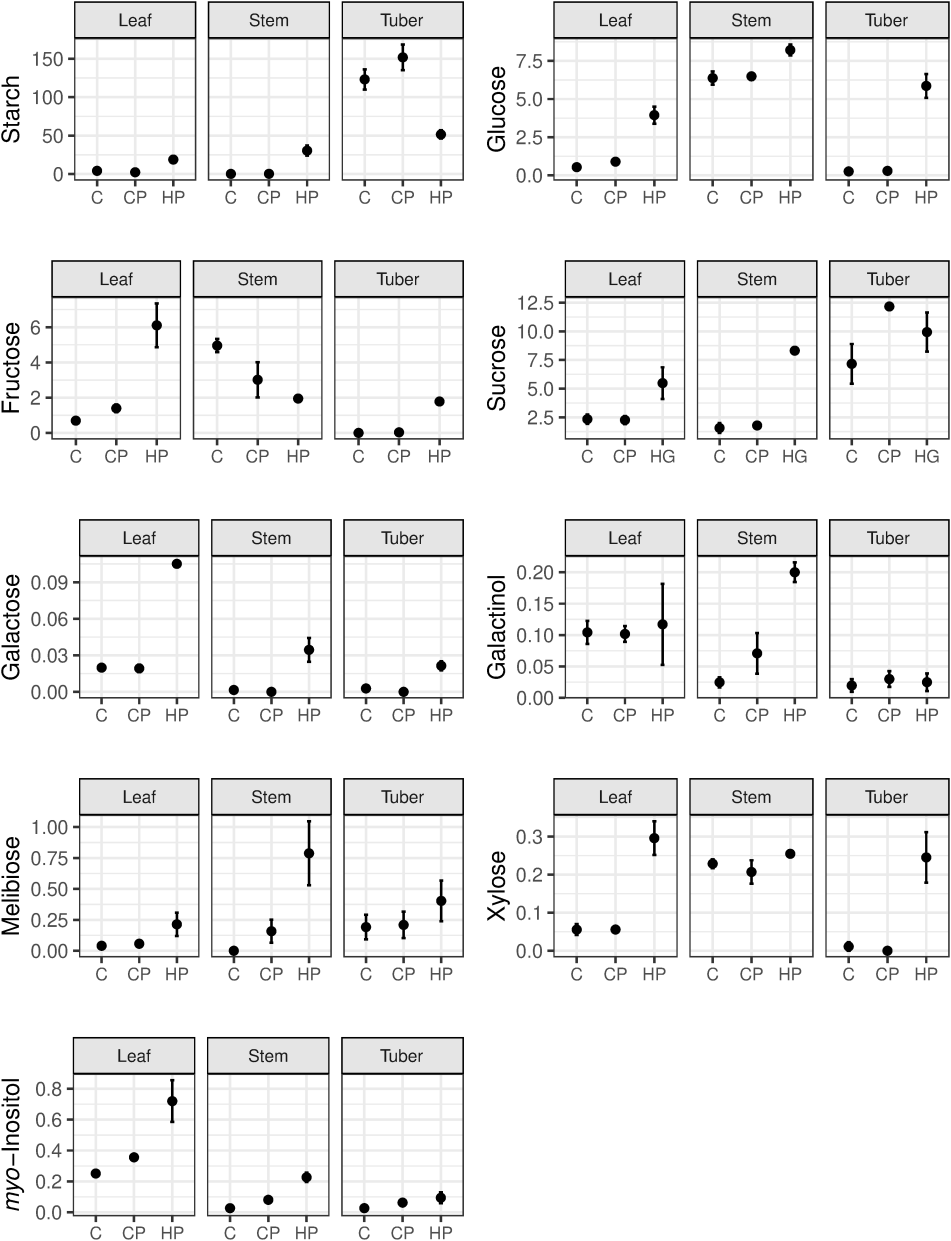
Mean concentrations of starch and sugars in potato plant tissues (mg g^-1^ fresh weight) harvested 7 weeks post-treatment. Error bars represent the standard error of the mean. C = control, CP = uninfected tomato potato psyllid (TPP), HP = TPP +* *‘*Candidatus* Liberibacter solanacearum’ (Lso).

Starch, glucose and fructose concentrations showed major changes in response to Lso infection (Fig 7). Starch was elevated > 4-fold in HP leaf and stem samples compared to uninfected (C and CP) samples (*p* < 0.05), and decreased > 2-fold in HP tuber samples compared with uninfected samples (*p* < 0.01). Glucose concentration was increased in all three tissues in HP samples relative to uninfected samples, with a > 4 fold increase in leaf (p < 0.01) and a > 10 fold increase in tuber (*p* < 0.001). Fructose followed a similar pattern to glucose in leaf and tuber tissues but showed a decrease in infected stems (*p* < 0.05). An increase in sucrose concentration (> 2-fold) was evident in HP stem samples (*p* < 0.001) but changes in leaf and stem were not significant. Galactose, galactinol, melibiose, xylose and *myo*-inositol were present at much lower concentrations and were detected in all tissues. The concentrations of these sugars showed increases in HP samples in response to Lso infection in some of the three tissues (*p* < 0.05). Galactose was increased in all three tissues (*p* < 0.01), whereas galactinol and melibiose were only significantly increased in stem (*p* < 0.05). Xylose showed increases in leaf and tuber (*p* < 0.01), whereas *myo*-inositol was increased in leaf and stem (*p* < 0.05). Raffinose, adonitol, melezitose, planteose, trehalose and stachyose were also detected in some or all tissues, but did not show any significant differences between treatments (*p* > 0.05).

The mean soluble acid invertase showed increased activity in HP tuber samples compared to C and CP samples, whereas leaf and stem samples showed increased activity in CP and HP compared to C. However, only the increase in CP stem tissue was significant (*p* > 0.05, S8 Fig).

### Metabolomic analysis of potato plants infected with Lso

Untargeted metabolomics analysis yielded four data sets, each comprising 2400–3000 mass features, with considerable overlap between sets (S9 Table). For each set, 17–58% of the mass features were tentatively identified.

**Fig 8:**
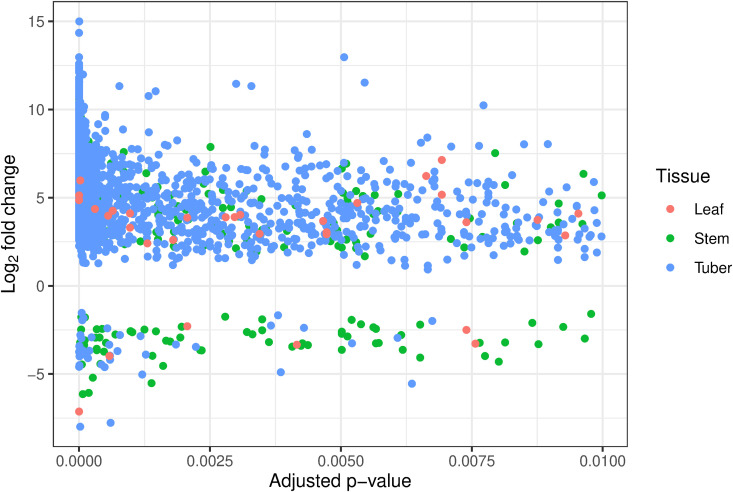
Mass features showing differences in concentration between HP and CP potato samples. The four data sets are combined, so there may be duplication of some metabolites where they were observed in more than one set. Both identified and unidentified mass features are included.

To identify metabolites which showed altered concentration in response to Lso infection, metabolomics data from HP and CP samples were compared. Many mass features showed significantly altered concentrations in the tuber (padj < 0.01), and far fewer in the stem and leaf. In all three tissues, there were many more mass features that showed increased concentration, rather than decreased concentration, in response to infection (Fig 8).

**Fig 9:**
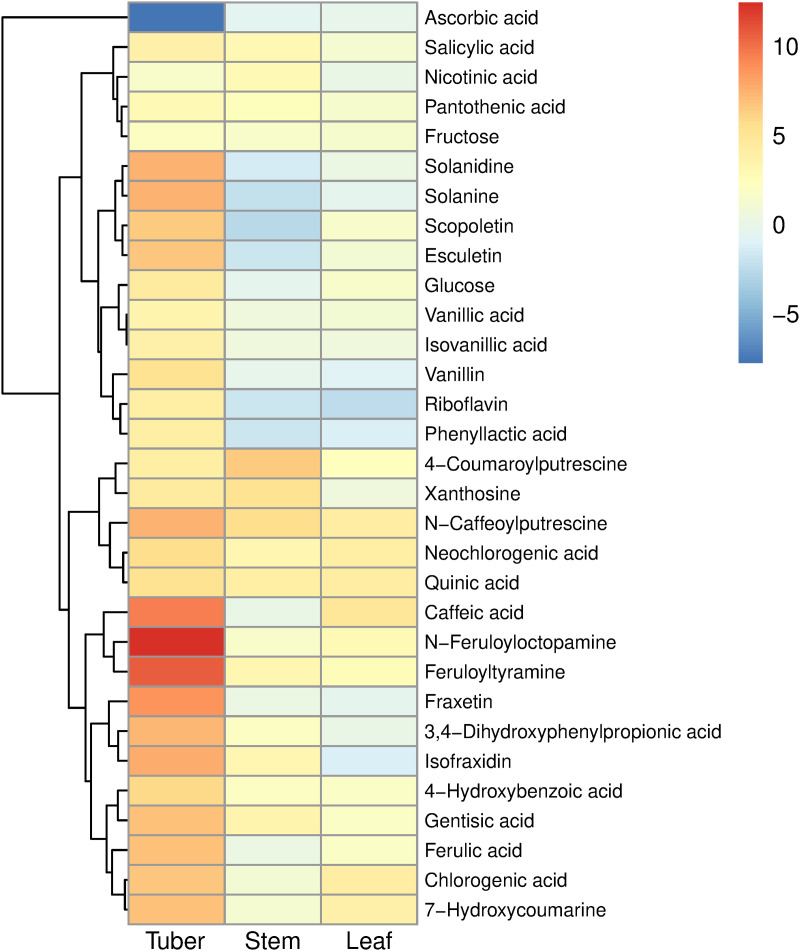
Heatmap of selected compounds which showed significant changes in concentration in potato tissues in response to ‘*Candidatus* Liberibacter solanacearum’ (Lso) infection (padj < 0.01), based on HP (tomato potato psyllid infected with ‘*Candidatus* Liberibacter solanacearum’) vs CP (uninfected tomato potato psyllid) treatments. Colour indicates log_2_ fold change.

In tuber tissue, the metabolites showing increased concentrations included several compounds produced via the phenylpropanoid pathway: hydroxycinnamic acids (caffeic acid, ferulic acid, chlorogenic acids), hydroxycinnamic acid amides (N-feruloyloctopamine, feruloyltyramine, N-caffeoylputrescine) and coumarins (fraxetin, isofraxidin, esculetin, scopoletin) (Fig 9). Hydroxybenzoic acids (salicylic acid (SA), gentisic acid, vanillic acid) and derivatives such as vanillin were also increased during Lso infection. There were also increased concentrations of the steroidal alkaloids solanine and solanidine. Other compounds that were increased were quinic acid, phenyllactic acid, riboflavin (vitamin B2), pantothenic acid (vitamin B5), and xanthosine. Glucose and fructose showed increases too, but sucrose was not significantly changed, consistent with the carbohydrate results presented above. The greatest decrease in concentration was ascorbic acid (log_2_fold change = −7.7). Of the 16 amino acids that were observed, only isoleucine showed a significant change (increase) in concentration.

Some of the compounds which were increased by the presence of Lso in stem were the hydroxycinnamic acid amide 4-coumaroylputrescine (which did not show an altered concentration in the tuber), and hydroxybenzoic acids SA and gentisic acid, which were also increased in tuber tissue. Increases were also observed in pantothenic acid (vitamin B5), nicotinic acid (vitamin B3), and xanthosine.

There were also many unidentified compounds which showed significant increases and decreases in concentration in response to Lso infection, in all three tissues (S9 Table).

## Discussion

Many of the changes observed in potato plants in response to infection with Lso were typical generalized disease responses, including redox and stress responses, and changes in carbohydrate and phenylpropanoid metabolism. In particular, concentrations of the phytohormone SA, which has a critical role in signalling plant defence responses [45], were increased in both stem and tuber samples. However these defence responses have not been sufficient to provide resistance to the disease. Accordingly, a recent publication reported that gene edited deletions in the salicylate receptor gene *StNPR3* (a negative regulator of SA defences) conferred resistance to ZC [46] by stimulating SA signalling.

While all three tissues showed substantial changes in metabolism and gene expression, indicating that the bacterial infection had a systemic effect on the whole plant, the effect on the tuber was the most prominent.

### Effects of Lso infection on potato tuber

**Fig 10:**
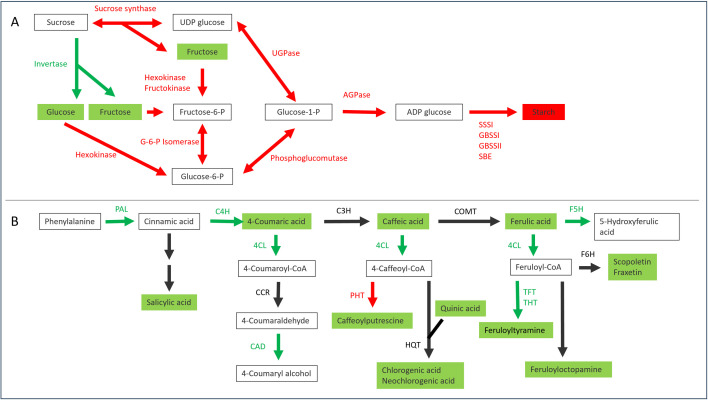
Integration of transcriptomic and metabolomic data highlighted the extensive changes in the starch synthesis (A) and phenylpropanoid (B) pathways in potato tuber infected with ‘*Candidatus* Liberibacter solanacearum’. Metabolites are shown in boxes; text adjacent to arrows represent genes that encode enzymes; green represents an increase in gene expression or metabolite concentration; red indicates a decrease; black is no change or inconclusive.

The most profound response was the comprehensive depression of the starch synthesis pathways in the tuber, in which all the genes (or gene families) involved in the pathways from sucrose to starch, with the exception of invertases, showed decreased expression (Figs 3, 10A). The vacuolar invertase gene showed increased expression, while the gene encoding Kunitz-type tuber invertase inhibitor, which is highly expressed in healthy tubers, had decreased expression. However, the vacuolar invertase inhibitor StInvInh2B (PGSC0003DMT400011760) [47,48] was not differentially expressed in tuber tissue. Both these inhibitors are implicated in regulation of vacuolar invertase [49]. The β subunit of SnRK1, which blocks StInvInh2B activity [48], had increased expression. These combined changes in gene expression would be expected to produce an increase in invertase activity. The tuber tissues showed decreased starch and increased glucose and fructose (Figs 7, 10A), although the increase in invertase activity in HP tuber samples was not significant. It appears that the disease is not preventing sucrose from reaching the tuber, but is redirecting sucrose away from starch synthesis and towards glucose and fructose, perhaps as a source of nutrition for Lso. This may represent manipulation of plant metabolism for the benefit of Lso. The gene models predicted from the Lso genome sequence indicate that Lso has a gene encoding a glucose/galactose transporter [50] but nothing with which to import fructose or sucrose. Galactose is also increased in the Lso-infected tuber, providing another potential Lso food source. An increased galactose concentration is potentially toxic to the potato plants and could inhibit growth [51]. Tuber tissues also showed increased accumulation of transcripts from genes involved in RFO synthesis (Fig 3), but no increase in the RFOs they produce. Several SWEET genes showed increased expression in the Lso-infected tuber, particularly those encoding putative transporters of sucrose [52]. SWEET genes are induced during infections by other bacterial pathogens and may be a means by which plants are exploited for pathogen nutritional gain [53].

The 16 amino acids which were identified in the tuber did not show significant changes in response to Lso infection, with the exception of isoleucine. Previous studies have shown an overall trend of increased amino acid concentrations in response to Lso infection; however, there was considerable variation among cultivars, with duration of infection, and in which amino acids were affected [3,14,15,54]. As the increases in amino acid concentrations were not substantial, the increased discolouration observed when infected tubers are fried may be more due to increased reducing sugars rather than amino acids.

Infected tubers also showed other changes relevant to the role of potato in human nutrition. Concentrations of the toxic glycoalkaloids solanine and solanidine were increased more than 100-fold (Fig 9, S9 Table), which potentially puts the concentration above the maximum acceptable limit of 20 mg/100 g fresh weight, and therefore not suitable for human consumption [55]. The concentration of ascorbate was decreased in infected tubers, as was the expression of genes involved in ascorbate metabolism (dehydroascorbate reductase, monodehydroascorbate reductase), although expression of the L-galactose dehydrogenase gene, involved in ascorbate biosynthesis, was increased (S1 Table). Because people eat large quantities of potato, it provides a significant source of vitamin C in the European diet [56]. These changes in glycoalkaloid and ascorbate content could have detrimental effects on the nutritional value of the product. However, there were also modest increases in pantothenic acid (vitamin B5) and riboflavin (vitamin B2) in the tuber (Fig 9). Nine of the 15 patatin genes showed decreased expression in tuber tissue, including some with high expression in growing tubers (S1 Table). As these are the major storage proteins in the tuber, this might be expected to decrease the overall protein content. Previous studies have produced conflicting results on this point, with reports of both increased protein content [16], and decreased protein content due to decreased concentrations of protease inhibitors [57]. Phenylpropanoids, which have antioxidant properties, are increased in infected tubers, which may improve the nutritional value of infected tubers [58]. In potato breeding, tolerance of new breeding lines to ZC disease is often measured by a frying test, but other assessments may be required to avoid breeding potatoes which have reduced Maillard reaction browning but have the potential for glycoalkaloid toxicity, or other undesirable changes, when infected with Lso.

The PAL enzyme performs the first step in the phenylpropanoid pathway and therefore initiates the biosynthesis of polyphenol compounds such as flavonoids, phenylpropanoids, and lignin in plants. This is one of the pathways most clearly affected by Lso infection, with significant increases in both gene expression and metabolites (Figs 4, 9, 10B). Changes in expression of genes associated with biosynthesis of HCAAs were also observed for both stem and tuber, and corresponding alterations to the HCAA metabolite profiles occurred for these tissues. Infection of potato plants with late blight caused similar changes in HCAA content and HCAA biosynthetic gene expression [59]. HCAAs are phytoalexins, have antimicrobial properties, and have a role in tuber formation [60,61].

The linolenic acid and JA pathways are altered in ZC disease (Fig 5). Expression of genes involved in producing linolenic acid in the chloroplast were decreased in the tuber, while those downstream in the pathway producing JA in the peroxisome, and methyl jasmonate and JA-isoleucine in the cytoplasm, were increased. These compounds have roles in defence and development, with JA involved in regulation of defence-related genes [62]. However, the concentration of JA was not significantly altered in any tissue.

GO analysis showed that the biggest changes in tuber gene expression concerned the microtubules (S7 Table). This could be due to the role of microtubules in the mitotic spindle, and a reduction of mitosis related to the slow growth rate of infected tubers. However, microtubules also have important roles in defence, immune responses and signalling. Some bacterial phytopathogens have effectors that target microtubules, to overcome plant defences [63].

Previous studies of ZC disease found increased activity of several enzymes in diseased tubers, specifically PPO, peroxidase and glucanase activities [3,14]. The increase in PPO activity would account for the increased enzymatic browning of cut surfaces of raw infected tubers that was observed in both our study and others [1]. However, the enzyme assay results from the other studies contrast with our transcriptomic results, which found that the accumulation of transcripts from the genes encoding these enzymes predominantly decreased (S1 Table). Similarly PPO and peroxidase genes were found to be down-regulated in infected tubers [18]. The increased enzymatic browning and decreased PPO gene expression can perhaps be reconciled by the metabolomics results that showed that substrates of PPO were increased (chlorogenic acid, caffeic acid, ferulic acid, Fig 9), and the antioxidant ascorbate was decreased. PPO activity has been associated with increased disease resistance in potato [64].

Some of the transcriptomic changes that result from Lso infection may be associated with slower tuber growth rather than a direct response to the pathogen. For example, decreased expression of genes associated with starch synthesis, patatins, cell walls and microtubules in the tuber may reflect the reduced growth rate of Lso-infected tubers. Most of the protein in tubers is stored in the vacuole and consists of protease inhibitors, patatins, and lipoxygenases [65]. Genes encoding these proteins include some of those with the highest expression in healthy growing tubers, which showed decreased expression in response to Lso. One of these genes (PGSC0003DMT400054145) encodes the 9S-lipoxygenase highly expressed in healthy tuber which has been associated with regulation of tuber enlargement [66].

### Response of potato stem tissue to Lso infection

It has been suggested that infection with Lso causes stem tissues to take on some characteristics of tubers, such as accumulation of starch and the tuber storage proteins, patatins [16]. Our results showed an increase in starch in the stem (Fig 7), and also increased expression of three patatin genes in stem tissue (S1 Table) which supports this idea. Stem samples also showed an increased concentration of both galactinol (Fig 7) and galactinol synthase gene expression (Fig 3), but the same was not true for RFOs and their biosynthetic genes. Galactinol and RFOs have roles in protection from osmotic and oxidative stress, and responses to pathogens, and RFOs are used as to transport carbohydrate in some plants [67] but not potato [68].

GO groups associated with hormones and sterols were also enriched in genes showing increased expression in stems (S7 Table). Four of the genes showing the greatest increases in expression in stem were involved in glycoalkaloid synthesis (Table 2, Fig 6). The gene showing the biggest change in stem tissue (ERF1, ethylene-responsive transcription factor,log_2_fold change = +10.6 HP vs CP) is also known as GAME 9, and has been shown to regulate the biosynthesis of steroidal alkaloids [44]. Several other genes involved in glycoalkaloid biosynthesis [69] also showed increased expression in stem tissue (Fig 6). However, the SMT1 gene, in the pathway from cycloartenol to sterols, had decreased expression in stem tissue. SMT1 overexpression was associated with reduced glycoalkaloids in potato leaves and tubers [70]. Therefore, decreased SMT1 expression and increased SSR2 expression in stems of Lso-infected plants may provide a switch which directs cycloartenol into the pathway to cholesterol and glycoalkaloids, rather than into sterols such as stigmasterol and brassinosteroids. However, increased glycoalkaloids were not seen in the stem, but were in the tuber (Fig 9). This could be explained by synthesis in the stem and transport to the tuber, but it is not clear whether this occurs [71]. Glycoalkaloids are desirable in the stem and leaf for protection from pests and pathogens but are not wanted in tuber tissue destined for human consumption.

Several of the genes showing most decreased expression encoded dirigent proteins (Table 2), which are involved in the stereochemistry of lignan synthesis. Lignans are phytoestrogens and antioxidants, are thought to be involved in defence against insect feeding, and are also products of the phenylpropanoid pathway. The stem metabolomics results revealed increases in 4-coumaroylputrescine, a product of the phenylpropanoid pathway, but not of the other HCAAs which were increased in tuber tissue (Fig 9). Most of the genes involved in the biosynthesis of these compounds did not show increased expression in the stem, the exceptions being ornithine decarboxylase and arginine decarboxylase, which are involved in putrescine biosynthesis (Fig 4). Both stem and tuber showed increased amounts of the purine alkaloid xanthosine, which is associated with production of reactive oxygen species for stress signalling and pathogen defence [72].

### Effects of Lso infection on leaf tissue

One of the major effects of ZC in leaf tissues was the down-regulation of genes involved in photosynthesis (S7 Table), in agreement with previous ZC transcriptomic reports [18,73], and a common response to disease. Reduction of photosynthetic rate has been reported for ZC [74]. This response is thought to be mediated by increases in hexose concentrations [75], which were also observed in leaf tissue in this study (Fig 7). Accumulation of starch in leaf tissues was also observed here, which agrees with previous reports [74]. Many heat shock protein genes had increased expression in leaf tissue, and were among the biggest fold changes in the leaf genes (Table 2, S1 Table). Heat shock proteins are molecular chaperones which ensure correct protein folding and translocation, and are important in responses to stress, including pathogens [76]. Genes involved in ABA signalling showed increased expression, including a dehydrin, TAS14 (Table 2), which is induced by ABA, stress and drought. ABA signalling is associated with resistance to abiotic stress, and has a complex role in plant-microbe interactions, where it often suppresses host immune responses but can also promote disease resistance [77]. Some of the most down-regulated genes in leaf tissues were SAUR family genes (Table 2), which have diverse roles in regulation of growth and development [78] but do not seem to have a direct role in defence. Other developmental genes were also down-regulated in the leaf, including three flowering locus T genes (S1 Table), which encode the phloem-mobile FT proteins that control flowering, tuberization, and other meristem-associated transitions [79]. One of these also showed decreased expression in Lso-infected tubers. LCMS revealed few secondary metabolite changes in leaf tissue despite there being changes in expression of many genes. However, the LCMS results only show a subset of all the metabolites present in a plant, which is dependent on the extraction protocol.

## Conclusions

ZC had a substantial effect on potato plant metabolism, particularly in the tuber. Carbohydrate metabolism was disrupted and many changes occurred in secondary metabolism pathways. The potato plant response to Lso infection included numerous typical disease responses. In this susceptible cultivar, these responses were providing partial protection from the disease, such that the plants survived long enough to produce tubers, but the tubers produced were very small, with insufficient yield and quality to satisfy industry requirements. Therefore, it is possible that a means of stimulating the defence responses could provide protection against the disease. While some of the effects of the disease are plant defence responses, other changes are probably instigated by the pathogen for its own benefit, such as nutritional gain, but the data presented here are unable to distinguish between the two. As the main problem that ZC causes for the potato industry is the browning associated with frying, which is related to concentrations of reducing sugars, breeding for disease tolerance may benefit from targeting genes encoding invertases, invertase inhibitors and the SnRK1 genes involved in regulation of their activity. Breeding for ZC tolerance will also need to consider the Lso-induced changes in secondary metabolites, particularly the toxic steroidal alkaloids. The response of a single susceptible cultivar to ZC tells us much about the effects of the disease at a molecular level; however, further research comparing susceptible and tolerant/resistant cultivars is required to further understand the molecular basis of tolerance/resistance, which can then inform breeding for tolerant/resistant cultivars.

## Supporting information

S1 Table Differentially expressed genes (DEGs) mentioned in text and figures. (XLSX)

S2 Table Zebra chip (ZC) disease symptoms and potato tuber size at time of sampling. (DOCX)

S3 Figure Quantification of ‘*Candidatus* Liberibacter solanacearum’ (Lso) in potato plant tissues by qPCR. (DOCX)

S4 Table Genes differentially expressed between HP and CP treatments in potato leaf. Genes were considered to be differentially expressed when padj < 0.001 and log_2_ fold change > |1|. CP = uninfected tomato potato psyllid (TPP), HP = TPP +* Candidatus* Liberibacter solanacearum (Lso).
(XLSX)

S5 Table Genes differentially expressed between HP and CP treatments in potato stem. Genes were considered to be differentially expressed when padj < 0.001 and log_2_ fold change > |1|. CP = uninfected tomato potato psyllid (TPP), HP = TPP + *Candidatus* Liberibacter solanacearum (Lso).
(XLSX)

S6 Table Genes differentially expressed between HP and CP treatments in potato tuber. Genes were considered to be differentially expressed when padj < 0.001 and log_2_ fold change > |1|. CP = uninfected tomato potato psyllid (TPP), HP = TPP + *Candidatus* Liberibacter solanacearum (Lso).
(XLSX)

S7 Table Potato gene ontology enrichment results significant at *p* < 0.01. (XLSX)

S8 Figure Soluble acid invertase enzyme activity (nmol gFW^-1^ min^-1^) in potato plant tissues. (DOCX)

S9 Table Mass features detected by untargeted metabolomics of potato tissues. (XLSX)

## References

[1] Munyaneza JE. Zebra Chip Disease of Potato: Biology, Epidemiology, and Management. Am J Pot Res. 2012;89(5):329–50. doi: 10.1007/s12230-012-9262-3

[2] Anderson JAD, Walker GP, Alspach PA, Jeram M, Wright PJ. Assessment of Susceptibility to Zebra Chip and *Bactericera cockerelli* of Selected Potato Cultivars under Different Insecticide Regimes in New Zealand. Am J Potato Res. 2012;90(1):58–65. doi: 10.1007/s12230-012-9276-x

[3] Wallis CM, Chen J, Civerolo EL. Zebra chip-diseased potato tubers are characterized by increased levels of host phenolics, amino acids, and defense-related proteins. Physiological and Molecular Plant Pathology. 2012;78:66–72.

[4] Pitman AR, Drayton GM, Kraberger SJ, Genet RA, Scott IAW. Tuber transmission of ‘*Candidatus* Liberibacter solanacearum’ and its association with zebra chip on potato in New Zealand. Eur J Plant Pathol. 2010;129(3):389–98. doi: 10.1007/s10658-010-9702-1

[5] Lin H, Gudmestad NC. Aspects of pathogen genomics, diversity, epidemiology, vector dynamics, and disease management for a newly emerged disease of potato: zebra chip. Phytopathology. 2013;103(6):524–37. doi: 10.1094/PHYTO-09-12-0238-RVW 23268582

[6] Vereijssen J, Smith GR, Weintraub PG. *Bactericera cockerelli* (Hemiptera: Triozidae) and *Candidatus* Liberibacter solanacearum in Potatoes in New Zealand: Biology, Transmission, and Implications for Management. Journal of Integrated Pest Management. 2018;9(1). doi: 10.1093/jipm/pmy007

[7] Gilkes JM, Frampton RA, Smith GR, Dobson RCJ. Potential pathogenicity determinants in the genome of ‘*Candidatus* Liberibacter solanacearum’, the causal agent of zebra chip disease of potato. Australasian Plant Pathology. 2018;47(2):119–34.

[8] Wang N, Pierson EA, Setubal JC, Xu J, Levy JG, Zhang Y, et al. The *Candidatus* Liberibacter-host interface: insights into pathogenesis mechanisms and disease control. Annual Review of Phytopathology. 2017;55:451–82. 10.1146/annurev-phyto-080516-035513 28637377

[9] Buchman JL, Fisher TW, Sengoda VG, Munyaneza JE. Zebra chip progression: from inoculation of potato plants with Liberibacter to development of disease symptoms in tubers. American Journal of Potato Research. 2012;89(2):159–68.

[10] Secor GA, Rivera VV, Abad JA, Lee I-M, Clover GRG, Liefting LW, et al. Association of “*Candidatus* Liberibacter solanacearum” with Zebra Chip Disease of Potato Established by Graft and Psyllid Transmission, Electron Microscopy, and PCR. Plant Dis. 2009;93(6):574–83. doi: 10.1094/PDIS-93-6-0574 30764398

[11] Beard SS, Pitman AR, Kraberger S, Scott IAW. SYBR Green real-time quantitative PCR for the specific detection and quantification of ‘*Candidatus* Liberibacter solanacearum’ in field samples from New Zealand. Eur J Plant Pathol. 2012;136(1):203–15. doi: 10.1007/s10658-012-0156-5

[12] Li W, Abad JA, French-Monar RD, Rascoe J, Wen A, Gudmestad NC, et al. Multiplex real-time PCR for detection, identification and quantification of “*Candidatus* Liberibacter solanacearum” in potato plants with zebra chip. J Microbiol Methods. 2009;78(1):59–65. doi: 10.1016/j.mimet.2009.04.009 19409423

[13] Rashed A, Workneh F, Paetzold L, Gray J, Rush CM. Zebra Chip Disease Development in Relation to Plant Age and Time of “*Candidatus* Liberibacter solanacearum” Infection. Plant Dis. 2014;98(1):24–31. doi: 10.1094/PDIS-04-13-0366-RE 30708584

[14] Rashed A, Wallis CM, Paetzold L, Workneh F, Rush CM. Zebra chip disease and potato biochemistry: tuber physiological changes in response to ‘*Candidatus* Liberibacter solanacearum’ infection over time. Phytopathology. 2013;103(5):419–26. 23425237 10.1094/PHYTO-09-12-0244-R

[15] Rashed A, Wallis CM, Workneh F, Paetzold L, Rush CM. Variations in Zebra Chip Disease Expression and Tuber Biochemistry in Response to Vector Density. Phytopathology. 2016;106(8):854–60. doi: 10.1094/PHYTO-01-16-0026-R 27111802

[16] Alvarado VY, Odokonyero D, Duncan O, Mirkov TE, Scholthof HB. Molecular and physiological properties associated with zebra complex disease in potatoes and its relation with *Candidatus* Liberibacter contents in psyllid vectors. PLoS One. 2012;7(5):e37345. doi: 10.1371/journal.pone.0037345 22615987 PMC3355140

[17] Levy J, Ravindran A, Gross D, Tamborindeguy C, Pierson E. Translocation of “*Candidatus* Liberibacter solanacearum”, the Zebra Chip pathogen, in potato and tomato. Phytopathology. 2011;101(11):1285–91. doi: 10.1094/PHYTO-04-11-0121 21770778

[18] Nwugo CC, Sengoda VG, Tian L, Lin H. Characterization of physiological and molecular processes associated with potato response to Zebra chip disease. Hortic Res. 2017;4:17069. doi: 10.1038/hortres.2017.69 29238599 PMC5717366

[19] Vereijssen J. Ecology and management of *Bactericera cockerelli* and *Candidatus* Liberibacter solanacearum in New Zealand. Journal of Integrative Agriculture. 2020;19(2):333–7. doi: 10.1016/s2095-3119(19)62641-9

[20] Mora V, Ramasamy M, Damaj MB, Irigoyen S, Ancona V, Avila CA, et al. Identification and Characterization of Potato Zebra Chip Resistance Among Wild Solanum Species. Front Microbiol. 2022;13:857493. doi: 10.3389/fmicb.2022.857493 35966647 PMC9363700

[21] Prager SM, Cohen A, Cooper WR, Novy R, Rashed A, Wenninger EJ, et al. A comprehensive review of zebra chip disease in potato and its management through breeding for resistance/tolerance to “*Candidatus* Liberibacter solanacearum” and its insect vector. Pest Manag Sci. 2022;78(9):3731–45. doi: 10.1002/ps.6913 35415948

[22] Anderson JAD, Wright PJ, Jaksons P, Puketapu AJ, Walker GP. Assessment of tolerance to zebra chip in potato breeding lines under different insecticide regimes in New Zealand. American Journal of Potato Research. 2018;95:504–12.

[23] Rubio-Covarrubias OA, Cadena-Hinojosa MA, Prager SM, Wallis CM, Trumble JT. Characterization of the Tolerance against Zebra Chip Disease in Tubers of Advanced Potato Lines from Mexico. Am J Potato Res. 2017;94(4):342–56. doi: 10.1007/s12230-017-9570-8

[24] Craig R, Ye J, Rommens C. Use of invertase silencing in potato to minimize losses from zebra chip and sugar ends. 2013. https://patents.google.com/patent/WO2014074990A1/en#patentCitations

[25] Thompson SM, Johnson CP, Lu AY, Frampton RA, Sullivan KL, Fiers MWEJ, et al. Genomes of “*Candidatus* Liberibacter solanacearum” Haplotype A from New Zealand and the United States Suggest Significant Genome Plasticity in the Species. Phytopathology. 2015;105(7):863–71. doi: 10.1094/PHYTO-12-14-0363-FI 25822188

[26] Zhong S, Joung JG, Zheng Y, Chen YR, Liu B, Shao Y, et al. High-throughput illumina strand-specific RNA sequencing library preparation. Cold Spring Harbour Protocols. 2011;2011(8):940–9. 10.1101/pdb.prot5652 21807852

[27] Andrews S. FastQC: a quality control tool for high throughput sequence data. 2010 http://www.bioinformatics.babraham.ac.uk/projects/fastqc.

[28] Wingett SW, Andrews S. FastQ Screen: A tool for multi-genome mapping and quality control. F1000Res. 2018;7:1338. doi: 10.12688/f1000research.15931.2 30254741 PMC6124377

[29] Bolger AM, Lohse M, Usadel B. Trimmomatic: a flexible trimmer for Illumina sequence data. Bioinformatics. 2014;30(15):2114–20. doi: 10.1093/bioinformatics/btu170 24695404 PMC4103590

[30] Sharma SK, Bolser D, de Boer J, Sønderkær M, Amoros W, Carboni MF, et al. Construction of reference chromosome-scale pseudomolecules for potato: integrating the potato genome with genetic and physical maps. G3 (Bethesda). 2013;3(11):2031–47. doi: 10.1534/g3.113.007153 24062527 PMC3815063

[31] Potato Genome Sequencing Consortium. Potato Genome Sequencing Consortium Public Data Release 2011. http://solanaceae.plantbiology.msu.edu/pgsc_download.shtml.

[32] Potato Genome Sequencing Consortium, Xu X, Pan S, Cheng S, Zhang B, Mu D, et al. Genome sequence and analysis of the tuber crop potato. Nature. 2011;475(7355):189–95. doi: 10.1038/nature10158 21743474

[33] Anders S, Pyl PT, Huber W. HTSeq--a Python framework to work with high-throughput sequencing data. Bioinformatics. 2015;31(2):166–9. doi: 10.1093/bioinformatics/btu638 25260700 PMC4287950

[34] Love MI, Huber W, Anders S. Moderated estimation of fold change and dispersion for RNA-seq data with DESeq2. Genome Biol. 2014;15(12):550. doi: 10.1186/s13059-014-0550-8 25516281 PMC4302049

[35] Kanehisa M, Sato Y, Furumichi M, Morishima K, Tanabe M. New approach for understanding genome variations in KEGG. Nucleic Acids Res. 2019;47(D1):D590–5. doi: 10.1093/nar/gky962 30321428 PMC6324070

[36] Alexa A, Rahnenfuhrer J. Gene set enrichment analysis with topGO. https://bioconductor.org/packages/release/bioc/html/topGO.html. 2016.

[37] Amar D, Frades I, Danek A, Goldberg T, Sharma SK, Hedley PE, et al. Evaluation and integration of functional annotation pipelines for newly sequenced organisms: the potato genome as a test case. BMC Plant Biol. 2014;14:329. doi: 10.1186/s12870-014-0329-9 25476999 PMC4274702

[38] Gibon Y, Blaesing OE, Hannemann J, Carillo P, Höhne M, Hendriks JHM, et al. A Robot-based platform to measure multiple enzyme activities in Arabidopsis using a set of cycling assays: comparison of changes of enzyme activities and transcript levels during diurnal cycles and in prolonged darkness. Plant Cell. 2004;16(12):3304–25. doi: 10.1105/tpc.104.025973 15548738 PMC535875

[39] Nardozza S, Boldingh HL, Osorio S, Höhne M, Wohlers M, Gleave AP, et al. Metabolic analysis of kiwifruit (*Actinidia deliciosa*) berries from extreme genotypes reveals hallmarks for fruit starch metabolism. J Exp Bot. 2013;64(16):5049–63. doi: 10.1093/jxb/ert293 24058160 PMC3830485

[40] Smith GS, Clark CJ, Boldingh HL. Seasonal Accumulation of Starch by Components of the Kiwifruit Vine. Annals of Botany. 1992;70(1):19–25. doi: 10.1093/oxfordjournals.aob.a088434

[41] R Core Team. R: A language and environment for statistical computing. Vienna, Austria: R Foundation for Statistical Computing. 2018.

[42] Lenth R. Emmeans: Estimated marginal means, aka least-squares means. https://CRAN.R-project.org/package=emmeans. 2020.

[43] Hope RM. Rmisc: Ryan miscellaneous. 2013.

[44] Cardenas PD, Sonawane PD, Pollier J, Vanden Bossche R, Dewangan V, Weithorn E, et al. GAME9 regulates the biosynthesis of steroidal alkaloids and upstream isoprenoids in the plant mevalonate pathway. Nature Communications. 2016;7:10654. 10.1038/ncomms10654 PMC4756317 26876023

[45] Gao L, Tu ZJ, Millett BP, Bradeen JM. Insights into organ-specific pathogen defense responses in plants: RNA-seq analysis of potato tuber-Phytophthora infestans interactions. BMC Genomics. 2013;14:340. doi: 10.1186/1471-2164-14-340 23702331 PMC3674932

[46] Ramasamy M, Rajkumar MS, Bedre R, Irigoyen S, Berg-Falloure K, Kolomiets MV, et al. Genome editing of NPR3 confers potato resistance to *Candidatus* Liberibacter spp. Plant Biotechnol J. 2024;22(9):2635–7. doi: 10.1111/pbi.14378 38773935 PMC11331773

[47] Brummell DA, Chen RKY, Harris JC, Zhang H, Hamiaux C, Kralicek AV, et al. Induction of vacuolar invertase inhibitor mRNA in potato tubers contributes to cold-induced sweetening resistance and includes spliced hybrid mRNA variants. J Exp Bot. 2011;62(10):3519–34. doi: 10.1093/jxb/err043 21393382 PMC3130176

[48] Lin Y, Liu T, Liu J, Liu X, Ou Y, Zhang H, et al. Subtle Regulation of Potato Acid Invertase Activity by a Protein Complex of Invertase, Invertase inhibitor, and sucrose nonfermenting1-related protein kinase. Plant Physiol. 2015;168(4):1807–19. doi: 10.1104/pp.15.00664 26134163 PMC4528764

[49] Liu X, Cheng S, Liu J, Ou Y, Song B, Zhang C, et al. The potato protease inhibitor gene, St-Inh, plays roles in the cold-induced sweetening of potato tubers by modulating invertase activity. Postharvest Biology and Technology. 2013;86:265–71.

[50] Lin H, Lou B, Glynn JM, Doddapaneni H, Civerolo EL, Chen C, et al. The complete genome sequence of “*Candidatus* Liberibacter solanacearum”, the bacterium associated with potato zebra chip disease. PLoS One. 2011;6(4):e19135. doi: 10.1371/journal.pone.0019135 21552483 PMC3084294

[51] Joersbo M, Jorgensen K, Brunsteldt J. A selection system for transgenic plants based on galactose as selective agent and a UDP-glucose:galactose-1-phosphate uridyltransferase gene as selective gene. Molecular Breeding. 2003;11:315–23.

[52] Manck-Götzenberger J, Requena N. Arbuscular mycorrhiza Symbiosis Induces a Major Transcriptional Reprogramming of the Potato SWEET Sugar Transporter Family. Front Plant Sci. 2016;7:487. doi: 10.3389/fpls.2016.00487 27148312 PMC4830831

[53] Chen L-Q, Hou B-H, Lalonde S, Takanaga H, Hartung ML, Qu X-Q, et al. Sugar transporters for intercellular exchange and nutrition of pathogens. Nature. 2010;468(7323):527–32. doi: 10.1038/nature09606 21107422 PMC3000469

[54] Wallis CM, Rashed A, Wallingford AK, Paetzold L, Workneh F, Rush CM. Similarities and differences in physiological responses to “*Candidatus* Liberibacter solanacearum” infection among different potato cultivars. Phytopathology. 2014;104(2):126–33. doi: 10.1094/PHYTO-05-13-0125-R 23941779

[55] Shakya R, Navarre DA. Rapid screening of ascorbic acid, glycoalkaloids, and phenolics in potato using high-performance liquid chromatography. J Agric Food Chem. 2006;54(15):5253–60. doi: 10.1021/jf0605300 16848503

[56] Davey MW, Montagu MV, Inze D, Sanmartin M, Kanellis A, Smirnoff N, et al. PlantL-ascorbic acid: chemistry, function, metabolism, bioavailability and effects of processing. J Sci Food Agric. 2000;80(7):825–60. doi: 10.1002/(sici)1097-0010(20000515)80:7<825::aid-jsfa598>3.0.co;2-6

[57] Kumar GNM, Knowles LO, Knowles NR. Zebra chip disease decreases tuber (*Solanum tuberosum L*.) protein content by attenuating protease inhibitor levels and increasing protease activities. Planta. 2015;242(5):1153–66. doi: 10.1007/s00425-015-2346-9 26092706

[58] Dwivedi SL, Upadhyaya HD, Chung I-M, De Vita P, García-Lara S, Guajardo-Flores D, et al. Exploiting Phenylpropanoid Derivatives to Enhance the Nutraceutical Values of Cereals and Legumes. Front Plant Sci. 2016;7:763. doi: 10.3389/fpls.2016.00763 27375635 PMC4891577

[59] Pushpa D, Yogendra KN, Gunnaiah R, Kushalappa AC, Murphy A. Identification of late blight resistance-related metabolites and genes in potato through nontargeted metabolomics. Plant Molecular Biology Reporter. 2013;32(2):584–95.

[60] Dobritzsch M, Lübken T, Eschen-Lippold L, Gorzolka K, Blum E, Matern A, et al. MATE Transporter-Dependent Export of Hydroxycinnamic Acid Amides. Plant Cell. 2016;28(2):583–96. doi: 10.1105/tpc.15.00706 26744218 PMC4790871

[61] Macoy DM, Kim WY, Lee SY, Kim MG. Biosynthesis, physiology, and functions of hydroxycinnamic acid amides in plants. Plant Biotechnology Reports. 2015;9(5):269–78.

[62] Ruan J, Zhou Y, Zhou M, Yan J, Khurshid M, Weng W, et al. Jasmonic Acid Signaling Pathway in Plants. Int J Mol Sci. 2019;20(10):2479. doi: 10.3390/ijms20102479 31137463 PMC6566436

[63] Li J, Staiger CJ. Understanding Cytoskeletal Dynamics During the Plant Immune Response. Annu Rev Phytopathol. 2018;56:513–33. doi: 10.1146/annurev-phyto-080516-035632 29975609

[64] Taranto F, Pasqualone A, Mangini G, Tripodi P, Miazzi MM, Pavan S, et al. Polyphenol Oxidases in Crops: Biochemical, Physiological and Genetic Aspects. Int J Mol Sci. 2017;18(2):377. doi: 10.3390/ijms18020377 28208645 PMC5343912

[65] Jørgensen M, Stensballe A, Welinder KG. Extensive post-translational processing of potato tuber storage proteins and vacuolar targeting. FEBS J. 2011;278(21):4070–87. doi: 10.1111/j.1742-4658.2011.08311.x 21851554

[66] Kolomiets MV, Hannapel DJ, Chen H, Tymeson M, Gladon RJ. Lipoxygenase is involved in the control of potato tuber development. Plant Cell. 2001;13(3):613–26. doi: 10.1105/tpc.13.3.613 11251100 PMC135504

[67] Sengupta S, Mukherjee S, Basak P, Majumder AL. Significance of galactinol and raffinose family oligosaccharide synthesis in plants. Front Plant Sci. 2015;6:656. doi: 10.3389/fpls.2015.00656 26379684 PMC4549555

[68] Hannah MA, Zuther E, Buchel K, Heyer AG. Transport and metabolism of raffinose family oligosaccharides in transgenic potato. J Exp Bot. 2006;57(14):3801–11. doi: 10.1093/jxb/erl152 17050641

[69] Sonawane PD, Pollier J, Panda S, Szymanski J, Massalha H, Yona M, et al. Plant cholesterol biosynthetic pathway overlaps with phytosterol metabolism. Nat Plants. 2016;3:16205. doi: 10.1038/nplants.2016.205 28005066

[70] Arnqvist L, Dutta PC, Jonsson L, Sitbon F. Reduction of cholesterol and glycoalkaloid levels in transgenic potato plants by overexpression of a type 1 sterol methyltransferase cDNA. Plant Physiol. 2003;131(4):1792–9. doi: 10.1104/pp.102.018788 12692338 PMC166935

[71] Ginzberg I, Tokuhisa JG, Veilleux RE. Potato steroidal glycoalkaloids: biosynthesis and genetic manipulation. Potato Research. 2008;52(1):1–15.

[72] Ma X, Wang W, Bittner F, Schmidt N, Berkey R, Zhang L, et al. Dual and Opposing Roles of Xanthine Dehydrogenase in Defense-Associated Reactive Oxygen Species Metabolism in Arabidopsis. Plant Cell. 2016;28(5):1108–26. doi: 10.1105/tpc.15.00880 27152019 PMC4904670

[73] Levy JG, Mendoza A, Miller JC Jr, Tamborindeguy C, Pierson EA. Global gene expression in two potato cultivars in response to “*Candidatus* Liberibacter solanacearum” infection. BMC Genomics. 2017;18(1):960. doi: 10.1186/s12864-017-4313-2 29228896 PMC5725879

[74] Gao F, Zhao ZH, Jifon J, Liu TX. Impact of potato psyllid density and timing of infestation on zebra chip disease expression in potato plants. Plant Protection Science. 2016;52(4):262–9.

[75] Berger S, Sinha AK, Roitsch T. Plant physiology meets phytopathology: plant primary metabolism and plant-pathogen interactions. J Exp Bot. 2007;58(15–16):4019–26. doi: 10.1093/jxb/erm298 18182420

[76] Park C-J, Seo Y-S. Heat Shock Proteins: A Review of the Molecular Chaperones for Plant Immunity. Plant Pathol J. 2015;31(4):323–33. doi: 10.5423/PPJ.RW.08.2015.0150 26676169 PMC4677741

[77] Lievens L, Pollier J, Goossens A, Beyaert R, Staal J. Abscisic acid as pathogen effector and immune regulator. Frontiers in Plant Science. 2017;8:587. 28469630 10.3389/fpls.2017.00587 PMC5395610

[78] Ren H, Gray WM. SAUR Proteins as Effectors of Hormonal and Environmental Signals in Plant Growth. Mol Plant. 2015;8(8):1153–64. doi: 10.1016/j.molp.2015.05.003 25983207 PMC5124491

[79] Navarro C, Abelenda JA, Cruz-Oro E, Cuellar CA, Tamaki S, Silva J, et al. Control of flowering and storage organ formation in potato by FLOWERING LOCUS T. Nature. 2011;478(7367):119–22. 21947007 10.1038/nature10431

