# Supplementary material for: Transcriptomic and metabolomic profiling of the potato plant response to zebra chip disease
Source: PLoS One. 2025 Jul 9;20(7):e0328035. doi: 10.1371/journal.pone.0328035 (PMC12240308; doi:10.1371/journal.pone.0328035)
Supplement: S2 Table — (DOCX) [file pone.0328035.s002.docx]

**S2 Table. Zebra chip (ZC) disease symptoms and potato tuber size at time of sampling.**

| **Plant^a^** | **ZC symptoms in leaf** | **ZC symptoms in largest tuber** | **Length of largest tuber (mm)** |
| --- | --- | --- | --- |
| **C2** | Nil | Nil | 80 |
| **C4** | Nil | Nil | 47 |
| **C12** | Nil | Nil | 57 |
| **CP1** | Nil | Nil | 61 |
| **CP7** | Nil | Nil | 57 |
| **CP10** | Nil | Nil | 48 |
| **HP6** | Some leaves yellowing | Browning of vascular ring | 12 |
| **HP8** | Some leaves yellowing | Browning of vascular ring | 15 |
| **HP11** | Some leaves show yellow or purple colour | Browning of vascular ring | 30 |

^a^Plant prefixes indicate the treatment applied: C (control), CP (“cold psyllid”, uninfected tomato potato psyllid (TPP)), HP (“hot psyllid”, TPP infected with ‘*Candidatus* Liberibacter solanacearum’ (Lso).
