# Supplementary material for: Transcriptomic and metabolomic profiling of the potato plant response to zebra chip disease
Source: PLoS One. 2025 Jul 9;20(7):e0328035. doi: 10.1371/journal.pone.0328035 (PMC12240308; doi:10.1371/journal.pone.0328035)

**S3 Figure. Quantification of ‘*Candidatus* Liberibacter solanacearum’ (Lso) in potato plant tissues by qPCR.** Prefixes for sample names are: C (control), CP (uninfected tomato potato psyllid (TPP)), HP (TPP + Lso). Lso ratio = Lso copy number/EF1α copy number.


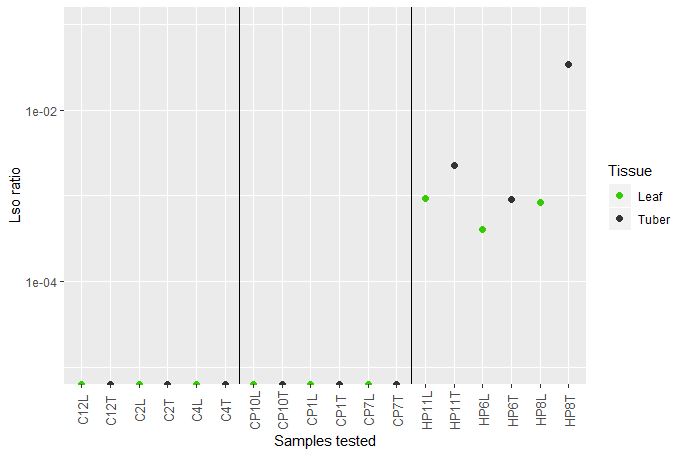

Supplement: S3 Figure — (DOCX) [file pone.0328035.s003.docx]
