# Supplementary material for: Transcriptomic and metabolomic profiling of the potato plant response to zebra chip disease
Source: PLoS One. 2025 Jul 9;20(7):e0328035. doi: 10.1371/journal.pone.0328035 (PMC12240308; doi:10.1371/journal.pone.0328035)

**S8 Figure . Soluble acid invertase enzyme activity (nmol gFW^-1^ min^-1^) in potato plant tissues.** Error bars represent the standard error of the mean. C = control, CP = uninfected tomato potato psyllid (TPP), HP = TPP + ‘*Candidatus* Liberibacter solanacearum’ (Lso), CG = graft with uninfected tomato plant, and HG = graft with Lso-infected tomato plant**.**


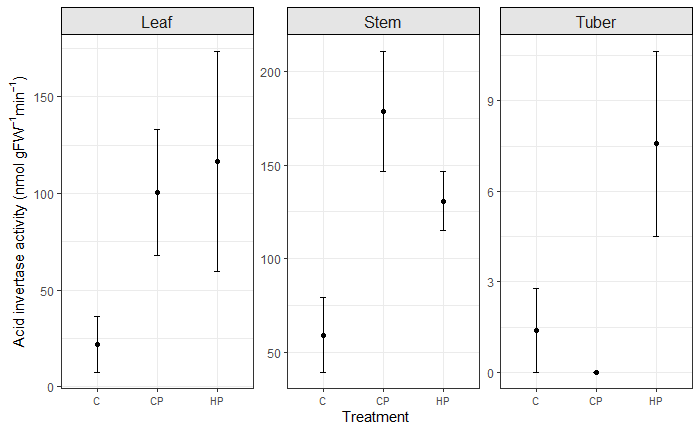

Supplement: S8 Figure — (DOCX) [file pone.0328035.s008.docx]
